# Meta-analyses including non-randomized studies of therapeutic interventions: a methodological review

**DOI:** 10.1186/s12874-016-0136-0

**Published:** 2016-03-22

**Authors:** Timor Faber, Philippe Ravaud, Carolina Riveros, Elodie Perrodeau, Agnes Dechartres

**Affiliations:** Centre d’Epidémiologie Clinique, Hôpital Hôtel-Dieu, APHP, Paris, France; Centre de Recherche Epidémiologie et Statistique, INSERM U1153, Paris, France; Faculté de Médecine, Université Paris Descartes, Sorbonne Paris Cité, Paris, France; Cochrane France, Paris, France; Department of Epidemiology, Mailman School of Public Health, Columbia University, New York, USA

**Keywords:** Meta-analyses, Therapeutic evaluation, Non-randomized studies, Reporting

## Abstract

**Background:**

There is an increasing number of meta-analyses including data from non-randomized studies for therapeutic evaluation. We aimed to systematically assess the methods used in meta-analyses including non-randomized studies evaluating therapeutic interventions.

**Methods:**

For this methodological review, we searched MEDLINE via PubMed, from January 1, 2013 to December 31, 2013 for meta-analyses including at least one non-randomized study evaluating therapeutic interventions. Etiological assessments and meta-analyses with no comparison group were excluded. Two reviewers independently assessed the general characteristics and key methodological components of the systematic review process and meta-analysis methods.

**Results:**

One hundred eighty eight meta-analyses were selected: 119 included both randomized controlled trials (RCTs) and non-randomized studies of interventions (NRSI) and 69 only NRSI. Half of the meta-analyses (*n* = 92, 49 %) evaluated non-pharmacological interventions. “Grey literature” was searched for 72 meta-analyses (38 %). An assessment of methodological quality or risk of bias was reported in 135 meta-analyses (72 %) but this assessment considered the risk of confounding bias in only 33 meta-analyses (18 %). In 130 meta-analyses (69 %), the design of each NRSI was not clearly specified. In 131 (70 %), whether crude or adjusted estimates of treatment effect for NRSI were combined was unclear or not reported. Heterogeneity across studies was assessed in 182 meta-analyses (97 %) and further explored in 157 (84 %). Reporting bias was assessed in 127 (68 %).

**Conclusions:**

Some key methodological components of the systematic review process—search for grey literature, description of the type of NRSI included, assessment of risk of confounding bias and reporting of whether crude or adjusted estimates were combined—are not adequately carried out or reported in meta-analyses including NRSI.

## Background

Randomized controlled trials (RCTs) are considered the gold standard for evidence-based medicine because they are designed to minimize the risk of bias [1]. However, the applicability of their results has been criticized because of restrictive selection criteria, with, commonly, exclusion of older adults and people with co-morbidities or severe disease [2–4]. Also, conducting an RCT is sometimes impossible or inappropriate (eg, when studying rare or long-term events) [1, 3, 5], which results in critical information gaps.

In contrast, observational studies, the overarching term for all non-experimental non-randomized studies (including cohort, case–control, and cross-sectional studies) [6], generally are more likely to reflect clinical practice in real life because of their broader range of participants, longer follow-up time, and lower costs than RCTs [7–10]. With the aim of generating evidence that will guide healthcare decisions, the field of comparative effectiveness research (CER) emphasizes the need to incorporate data from observational studies to complement RCTs [8, 11–16]. A comprehensive assessment in 2009 indicated that 54 % of CER studies had an observational study design [17]. Therefore, an increasing number of systematic reviews and meta-analyses are including data from non-randomized studies to assess therapeutic interventions.

Similar to systematic reviews of RCTs, reviews including non-randomized studies are expected to follow the general recommendations for good conduct, such as retrieving all relevant studies and assessing their risk of bias. However, some elements should be adapted specifically to the inclusion of non-randomized studies because their study designs inherently differ from RCTs [7, 9, 14, 18–23]. Lacking randomization, they are likely subject to confounding bias, which results in an imbalance in prognostic factors associated with the outcome of interest that may severely compromise the validity of their results [24].

Previous methodological reviews evaluating systematic reviews including observational studies exist [25–28]. However, these studies have a different objective. One assessed the main characteristics of all systematic reviews indexed in Medline on November 2004 whatever the type of included studies (ie, therapeutic, epidemiological, prognostic or diagnostic studies) [27]. Two others focused on the methods and reporting of harms in systematic reviews of adverse events [26, 28]. The last one was in the field of psychiatry and did not concern therapeutic evaluation but assessment of prevalence or association [25]. Further, none of these previous reviews has evaluated the specific methodological problems raised by the inclusion of non-randomized studies.

In this study, we performed a methodological review of meta-analyses including non-randomized studies of interventions (NRSI) to evaluate key methodological components common to all meta-analyses and those specifically related to the inclusion of non-randomized studies.

## Methods

### Study design

This is a methodological review of meta-analyses including NRSI for therapeutic evaluation. For clarity and consistency, we refer to this article as a “methodological review”, the systematic reviews with meta-analyses included in this methodological review as “meta-analyses”, and the studies included in these meta-analyses as “studies”.

### Search strategy

Our goal was not to create an exhaustive list of all meta-analyses that include NRSI but rather to identify a relatively representative sample of recently published meta-analyses that a health professional would most likely encounter when searching for meta-analyses. We therefore searched MEDLINE via PubMed because of its wide use among health professionals, combining keywords and MeSH terms for NRSI, systematic reviews, and meta-analyses (Appendix 1). The search was conducted on January 7, 2014 and restricted to the year 2013.

### Eligibility criteria

To be eligible, a meta-analysis had to examine a therapeutic or preventive intervention (such as vaccines) for efficacy or safety, include data from at least one NRSI, and be published in 2013. We excluded meta-analyses that included studies without a comparison group and meta-analyses of etiological assessment. When it was difficult to distinguish an etiological from a therapeutic evaluation, we agreed to include the former if the authors considered the inclusion of RCTs in their meta-analysis. To illustrate: a meta-analysis that investigated the association of the use of statins and risk of cancer would was considered a therapeutic evaluation if the authors planned to include RCTs. Individual patient data meta-analyses were also excluded, as were non-randomized studies that conducted a meta-analysis of the literature as secondary analysis. Finally, we did not include meta-analyses published in a language other than English or those for which the full text was not available.

### Selection of relevant meta-analyses

The selection of relevant meta-analyses was conducted in 2 steps. In the first step, one reviewer (CR) excluded clearly irrelevant studies based on the title, abstract, and full text, then, a second reviewer (TF) performed the final selection, discussing all doubtful cases with a third reviewer (AD).

### Data extraction

The data extraction form for this methodological review was developed from the MOOSE statement for reporting meta-analyses that include observational studies [29], the PRISMA statement for reporting systematic reviews and meta-analyses of studies evaluating healthcare interventions [30, 31], and the AMSTAR measurement tool for assessing the methodological quality of systematic reviews [32]. The data extraction form was tested by one reviewer (TF) with 10 studies before data extraction commenced.

Two reviewers (TF, CR) independently extracted all data in duplicate, resolving discrepancies with a third reviewer (AD) if necessary. The following characteristics were extracted from the full text and online appendix of each meta-analysis:

- *General characteristics:* We collected whether the journal was a specialty or a general journal, the location of the corresponding author, and the medical area. We verified whether the meta-analysis was registered on the international prospective register of systematic reviews by the University of York’s Centre for Reviews and Dissemination (PROSPERO). We collected whether epidemiologists or statisticians were involved, relying on the definition given by Delgado-Rodriguez et al.[33] and assessed whether the authors reported the funding sources and declared conflict of interests. We assessed whether the meta-analyses evaluated a pharmacological or non-pharmacological intervention. Non-pharmacological interventions were classified as surgical procedures or other interventions. We also assessed the type of studies included: only NRSI or both NRSI and RCTs.
- *Systematic review methods:*
  - ▪Search strategy: We collected how many and which electronic databases were searched, and whether the search strategy for at least one database was provided. We collected whether reference lists and journals were hand-searched and whether the authors searched for grey literature, and if yes, how: search of registries (eg, ClinicalTrials.gov), conference abstracts, or contacting experts. We assessed whether the authors restrict their searches by language.
  - ▪Study selection and data extraction process: We assessed whether study selection and data extraction were conducted in duplicate.
  - ▪Contact of the study authors: We noted whether it was mentioned that study authors were contacted for clarification or additional results.
  - ▪Methodological quality/risk of bias assessment: We assessed whether methodological quality or risk of bias assessment was conducted, what tools were used, and whether the assessment was conducted in duplicate.
- *Meta-analysis methods:*
  - ▪Studies combined: We assessed the types of NRSI included. NRSI were categorized as concurrent (prospective) cohort, nonconcurrent (retrospective) cohort, case–control, or historically controlled studies according to the definition by Ioannidis et al. [21]. We also assessed whether the authors combined the results from NRSI and RCTs and whether they combined results from different types of NRSI (eg, cohort and case–control studies).
  - ▪Meta-analysis model: We collected whether the authors used crude or adjusted estimates for NRSI and whether they used fixed- or random-effects models to pool the data. For adjusted estimates, we also collected whether the confounding factors taken into account were listed.
  - ▪Assessment and exploration of heterogeneity: We collected whether and how the authors assessed heterogeneity and whether they conducted meta-regression, subgroup, or sensitivity analyses to explore heterogeneity.
  - ▪Assessment of reporting bias: We collected information on whether the authors assessed reporting bias, and how.

### Statistical analysis

The analysis of the data consisted of descriptive statistics, providing numbers and percentages for qualitative variables and median (minimum, maximum, or interquartile range) for quantitative variables. The results were stratified for meta-analyses including only NRSI and those including both NRSI and RCTs. We did not assess statistical differences between these strata. Statistical analysis involved use of R 3.0.2. (R Core Team [2013]. R: A language and environment for statistical computing. R Foundation for Statistical Computing, Vienna, Austria. URL: http://www.R-project.org/).

## Results

### Study selection

**Fig. 1:**
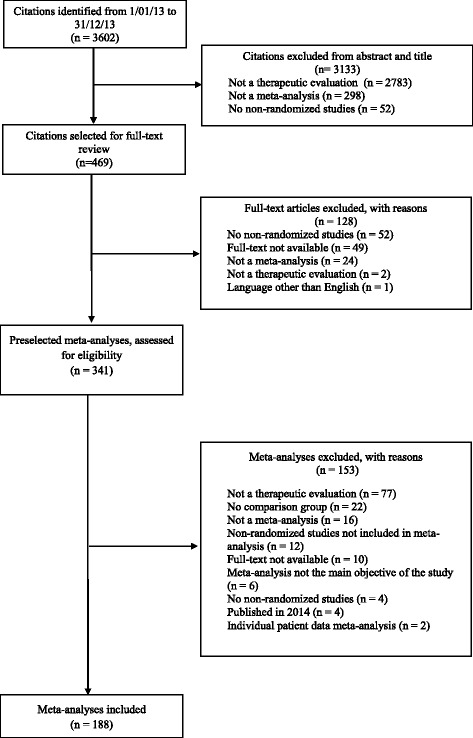
Study selection flowchart

Our MEDLINE search identified 3602 citations; Among the 341 potentially relevant meta-analyses, 188 were eligible for this review (Fig. 1). Complete references for the included meta-analyses and meta-analyses excluded are in Appendixes 2 and 3, respectively.

### General characteristics (Table 1)

**Table 1:** General characteristics of therapeutic meta-analyses published in 2013 and including non-randomized studies of intervention (*n* = 188)

Characteristic	Included NRSI only	Included NRSI&RCTs	All meta-analyses
	(*n* = 69)	(*n* = 119)	(*n* = 188)
	No (%)	No (%)	No (%)
Journal type			
Specialty	59 (86)	99 (83)	158 (84)
General	10 (14)	20 (17)	30 (16)
Location of corresponding author			
Asia	26 (38)	38 (32)	64 (34)
Europe	22 (32)	39 (33)	61 (32)
North-America	18 (26)	33 (28)	51 (27)
Other	3 (4)	9 (7)	12 (7)
Medical area			
Surgery	21 (30)	28 (24)	49 (26)
Cardiology	8 (12)	25 (21)	33 (18)
Oncology	6 (9)	19 (16)	25 (13)
Other	34 (49)	47 (39)	81 (43)
Type of intervention			
Pharmacological	36 (52)	56 (47)	92 (49)
Non-pharmacological	32 (46)	60 (50)	92 (49)
*Surgical procedures*	*30 (43)*	*44 (37)*	*74 (39)*
*Other*	*2 (3)*	*16 (13)*	*18 (10)*
Both pharmacological and non-pharmacological	1 (1)	3 (3)	4 (2)
Epidemiologists or statisticians involved	17 (25)	19 (16)	36 (19)
Declaration of conflict of interests	64 (93)	102 (86)	166 (88)
Potential conflict of interests	9 (13)	17 (14)	26 (14)
Funding source			
Public	30 (43)	36 (30)	66 (35)
No specific funding	10 (14)	29 (24)	39 (21)
Private	2 (3)	7 (6)	9 (5)
Both public and private	3 (4)	2 (2)	5 (3)
Not reported/unclear	24 (35)	45 (38)	69 (37)
Registration on PROSPERO	0	3 (3)	3 (2)

*NRSI* non-randomized studies of intervention; *RCTs* randomized controlled trials

Among the 188 included meta-analyses, 49 (26 %) were of surgery, 33 (18 %) cardiology, and 25 (13 %) oncology. Half of the meta-analyses assessed non-pharmacological interventions (*n* = 92, 49 %); 74 involved surgical procedures. Approximately one third (*n* = 69, 37 %) included only NRSI, and two thirds included both NRSI and RCTs (*n* = 119, 63 %).

In total, 36 meta-analyses (19 %) involved epidemiologists or statisticians. Conflict of interest was declared in 166 (88 %), with 26 reporting a potential conflict of interest. About one-third of the meta-analyses did not report a source of funding (*n* = 69, 37 %).

### Systematic review methods (Table 2)

**Table 2:** Systematic review methods of therapeutic meta-analyses published in 2013 and including non-randomized studies (*n* = 188)

Characteristic	Included NRSI only	Included NRSI&RCTs	All Meta-analyses
	(*n* = 69)	(*n* = 119)	(*n* = 188)
	No (%)	No (%)	No (%)
Search strategy			
No. of electronic databases searched, median [IQR]	3 [2–4]	3 [3, 4]	3 [3, 4]
> 2 databases searched	51 (74)	96 (81)	147 (78)
Electronic databases searched^a^			
Medline/PubMed/Ovid	69 (100)	118 (99)	187 (99)
EMBASE	52 (75)	97 (82)	149 (79)
Cochrane	45 (65)	81 (68)	126 (67)
Web of science/web of knowledge	14 (20)	22 (18)	36 (19)
Time-frame of search strategy reported	62 (90)	109 (92)	171 (91)
Time end of search-publication date (months), median [min-max]	10 [0–24]	9 [1–51]	10 [0–51]
Search strategy provided for at least one database	18 (26)	44 (37)	62 (33)
Searched reference lists for relevant studies	57 (83)	105 (88)	162 (86)
Hand searched journals	5 (7)	7 (6)	12 (6)
Searched for grey literature^a^	23 (33)	49 (41)	72 (38)
Conference abstracts	14 (20)	27 (23)	41 (22)
Registries (eg, ClinicalTrials.gov)	8 (12)	25 (21)	33 (18)
Experts	6 (9)	9 (8)	15 (8)
No restriction by language reported	20 (29)	62 (52)	82 (44)
Study selection			
Duplicate study selection	47 (68)	84 (71)	131 (70)
Methodological quality/risk of bias			
Assessment of MQ/RoB	47 (68)	88 (74)	135 (72)
Tools used for RCTs^a^			
Cochrane RoB Tool [41]	NA	42 (35)	NA
Jadad scale [42]	NA	22 (18)	NA
GRADE [43]	NA	9 (8)	NA
Other	NA	21 (18)	NA
Authors made their own tool	NA	3 (3)	NA
Unclear	NA	7 (6)	NA
Tools used for NRSI^a^			
Newcastle-Ottawa scale [44]	25 (36)	43 (36)	68 (36)
GRADE [43]	5 (7)	8 (7)	13 (7)
Cochrane RoB Tool [41]	3 (4)	7 (6)	10 (5)
Other tools	13 (19)	24 (20)	37 (20)
Authors made their own tool	6 (9)	6 (5)	12 (6)
Unclear	3 (4)	9 (8)	12 (6)
Confounding taken into account in risk of bias	13 (19)	20 (17)	33 (18)
Same tool used for RCTs and NRSI	NA	27 (23)	NA
Duplicate MQ/RoB assessment	30/47 (64)	57/88 (65)	87/135 (64)
Data extraction			
Duplicate data extraction	52 (75)	86 (72)	138 (73)
Contact authors for clarification or additional results	18 (26)	43 (36)	61 (32)

Data are no. (%), unless indicated. *IQR* interquartile range; *MQ* methodological quality; *NRSI* non-randomized studies of interventions; *RCTs* randomized controlled trials; *Cochrane RoB* Cochrane risk of bias tool; *NA* Not applicable

^a^More than one item could have been reported for each meta-analysis

#### Literature search

Overall, all but one of the meta-analyses reported the search of at least 1 electronic database and 147 (78 %) reported the search of > 2 electronic databases. One third provided the search strategy for each database (*n* = 62, 33 %). MEDLINE, Embase, and the Cochrane Library were most frequently searched (187 [99 %], 149 [79 %], and 126 (67 %) meta-analyses, respectively). In addition to the search of electronic databases, 162 meta-analyses (86 %) reported screening the reference lists of included studies, and 12 (6 %) reported hand-searching journals. About one-third of the meta-analyses (*n* = 72, 38 %) reported searching for grey literature: 41 (22 %) conference abstracts, 33 (18 %) registries, and 15 (8 %) contacted experts. For 82 meta-analyses (44 %), the authors reported that they did not restrict their searches by language.

#### Methodological quality/risk of bias assessment

Methodological quality or risk of bias of included studies was assessed in 135 (72 %) meta-analyses.

For the 119 meta-analyses including RCTs and NRSI, risk of bias was assessed in 88 (74 %) with 4 assessing risk of bias for RCTs only. RCTs were assessed with the Cochrane Risk of Bias tool in 42 (35 %) meta-analyses. The assessment of risk of bias involved the same tool for both RCTs and NRSI in 27 (23 %) meta-analyses. For the assessment of NRSI, a variety of tools were used. The most frequently used tool was the Newcastle-Ottawa Scale (*n* = 68). GRADE and the Cochrane Collaboration Risk of Bias Tool were used in 13 and 10 meta-analyses, respectively. In 37 meta-analyses, authors used other tools; in 12, they developed their own tools; and in 12, they were unclear about the methods used for assessing methodological quality/risk of bias. Overall, the authors have considered the risk of confounding bias in their risk of bias assessment in 33 meta-analyses (18 %). Of the 135 meta-analyses with an assessment of risk of bias, 87 (64 %) reported having performed it in duplicate.

### Meta-analysis methods (Table 3)

#### Studies combined

For 130 meta-analyses (69 %), the authors did not clearly report the design for each individual study. Among the meta-analyses that included both NRSI and RCTs (*n* = 119), for 88 (74 %), the results of NRSI and RCTs were combined.

**Table 3:** Meta-analysis methods of therapeutic meta-analyses published in 2013 and including non-randomized studies (*n* = 188)

Characteristic	Included NRSI only	Included NRSI&RCTs	All meta-analyses
	(*n* = 69)	(*n* = 119)	(*n* = 188)
	No (%)	No (%)	No (%)
Studies combined			
Type of studies included			
Only prospective cohort studies	2 (3)	3 (3)	5 (3)
Only cohort studies	18 (26)	34 (29)	52 (28)
Including also case–control studies	18 (26)	28 (23)	46 (24)
Including all types of NRSI	5 (7)	18 (15)	23 (12)
Other	28 (41)	39 (33)	67 (36)
“Observational studies”	6 (9)	22 (18)	28 (15)
“Prospective and retrospective studies”	11 (16)	12 (10)	23 (12)
“Retrospective studies”	11 (16)	5 (4)	16 (9)
Did not clearly report design for each study	43 (62)	87 (73)	130 (69)
Meta-analysis combining results of NRSI and RCTs	NA	88 (74)	NA
Results also presented separately	NA	44/88 (50)	NA
Meta-analysis combining studies of different designs	39 (57)	61 (51)	100 (53)
Meta-analysis model			
Network meta-analysis	0	2 (2)	2 (1)
Crude or adjusted estimates used			
Combined crude and adjusted estimates	10 (14)	12 (10)	22 (12)
Adjusted	8 (12)	13 (11)	21 (11)
Both crude and adjusted estimates separately	4 (6)	4 (3)	8 (4)
Crude	2 (3)	4 (3)	6 (3)
Not reported/unclear	45 (65)	86 (72)	131 (70)
If adjusted, list of confounding factors	15/22 (68)	19/29 (66)	34/51 (67)
Type of model			
Random-effects model	39 (57)	56 (47)	95 (50)
Fixed-effects model, unless high heterogeneity, then random-effects model	19 (28)	33 (28)	52 (28)
Both fixed- and random-effects models	5 (7)	21 (18)	26 (14)
Fixed-effects model	3 (4)	6 (5)	9 (5)
Not reported/unclear	3 (4)	3 (3)	6 (3)
Heterogeneity assessment & exploration			
Heterogeneity assessed^a^	66 (96)	116 (97)	182 (97)
I^2^	57 (83)	107 (90)	164 (87)
Cochran Q χ^2^ test	41 (59)	74 (62)	115 (61)
τ^2^	4 (6)	2 (2)	6 (3)
Heterogeneity explored^a^	54 (82)	103 (87)	157 (84)
Meta-regression analysis	13 (19)	21 (18)	34 (18)
Subgroup analysis	44 (64)	82 (69)	126 (67)
Sensitivity analysis	35 (51)	74 (62)	109 (58)
Reporting bias assessment^a^	49 (71)	78 (66)	127 (68)
Standard funnel plot	41 (59)	70 (59)	111 (59)
Egger’s test	32 (46)	36 (30)	68 (36)
Begg’s test	18 (26)	24 (20)	42 (22)
Among MAs with ≥10 studies, reporting bias assessed	24/29 (83)	58/76 (76)	82/105 (78)

*MAs* meta-analyses; *NRSI* non-randomized studies of intervention; *RCTs* randomized controlled trials

^a^More than one item could have been reported for each meta-analysis

Concerning NRSI combined, 52 meta-analyses (28 %) included only cohort studies and 5 only prospective cohort studies; 46 meta-analyses (24 %) combined cohort and case–control studies, and 23 (12 %) included all types of NRSI. The other 67 meta-analyses (36 %) included “observational studies” (without further details) (*n* = 28, 15 %), “prospective and retrospective studies” (*n* = 23, 12 %), and only “retrospective studies” (*n* = 16, 9 %).

#### Crude or adjusted estimates used for NRSI

For 131 meta-analyses (70 %), whether crude or adjusted estimates of treatment effect from the NRSI were used for the meta-analysis was unclear or not reported. For the remaining meta-analyses, the authors reported combining crude and adjusted estimates for 22 (12 %), only adjusted estimates for 21 (11 %), and only crude estimates for 6 (3 %). For 8 meta-analyses (4 %), the authors extracted both the crude and adjusted estimates and used them separately in 2 meta-analyses. Among the 51 meta-analyses involving adjusted estimates, 17 (33 %) did not report the confounding factors adjusted for.

#### Meta-analysis model

A random-effects model was used for half of the meta-analyses (*n* = 95). For 52 (28 %), a fixed-effects model was used primarily but then replaced with a random-effects model if high heterogeneity was observed in the model. For 26 meta-analyses (14 %), the authors used both fixed- and random-effects models, and for 9 (5 %), a fixed-effects model. The type of model was not reported or was unclear for 6 meta-analyses (3 %). We found 2 network meta-analyses (1 %).

#### Heterogeneity assessment

Almost all meta-analyses assessed heterogeneity (*n* = 182, 97 %). The I^2^ statistic was used in 164 meta-analyses (87 %), Cochran Q χ^2^ test in 115 (61 %), and between-study variance τ^2^ in 6 (3 %). Heterogeneity was explored in 157 meta-analyses (84 %) by subgroup analyses (*n* = 126, 67 %), sensitivity analyses (*n* = 109, 58 %) and meta-regression analyses (*n* = 34, 18 %).

For 44 of 88 (50 %) meta-analyses combining results from RCTs and NRSI, a subgroup or sensitivity analysis was based on the type of study (RCT vs NRSI). For 28 meta-analyses (15 %), subgroup or sensitivity analyses were based on the type of NRSI included.

#### Reporting bias assessment

Reporting bias was assessed in 127 meta-analyses (68 %) by standard funnel plots (*n* = 111, 59 %), Egger’s test (*n* = 68, 36 %), or Begg’s test (*n* = 42, 22 %). Overall, 82 of the 105 meta-analyses (78 %) including 10 or more studies reported having assessed reporting bias.

## Discussion

We systematically assessed key methodological components of a large sample of therapeutic meta-analyses including NRSI in a variety of medical areas. Our results highlight some important methodological shortcomings. Only 38 % of the meta-analyses reported having searched for grey literature. Specific points related to the inclusion of NRSI raise concerns, with 69 % of the meta-analyses not reporting the study design of the included NRSI, and 70 % not reporting whether crude or adjusted estimates were combined.

### Strengths and limitations of study

To the best of our knowledge, no previous study has comprehensively assessed both key methodological components common to all systematic reviews and elements specific to the inclusion of non-randomized studies. Other studies that previously evaluated methods or reporting of systematic reviews including NRSI concentrated on the reporting of harms [26, 28] and on systematic reviews in psychiatric epidemiology [25].

Our study has some limitations. The representativeness of our sample could be debated because we searched for studies in only one online database (MEDLINE), and limited our selection to meta-analyses in English. In addition, for the assessment of the methods, we depended completely on the reporting; we did not assess protocols or contact the authors if methods were not clearly reported. Even though poor reporting does not necessarily reflect poor conduct, it may severely limit the reader’s comprehension of the systematic review process [34].

Before being able to apply the results of any meta-analysis to patient care, health professionals need to evaluate the credibility of the methods of the meta-analysis [35]. One of the key methodological elements is the search for relevant studies. Because not all studies (and particularly those with negative results) are published in scientific journals, a meta-analysis must involve a search for grey literature to try to avoid such publication bias (a type of reporting bias) [24, 35]. However, we found that only 38 % of our meta-analyses reported having searched for grey literature. Because of no mandatory registration for NRSI as for RCTs, most NRSI are not registered, so searching for grey literature of NRSI is difficult [36]. However, a recent study found that for 32 % of the observational studies registered at ClinicalTrials.gov, unpublished results could be retrieved [37]. In contrast, we found that many meta-analyses assessed reporting bias (68 %). Reviewers may have compensated for the absence of searching for grey literature by assessing reporting bias. Evaluating reporting bias does not exempt the reviewers from searching for grey literature because the assessment of Funnel plot asymmetry may be subjective and statistical methods to test for asymmetry of the plot may lack power [38, 39].

Another critical part of the systematic review process is assessing the methodological quality or risk of bias of the studies included, because the validity of the meta-analysis could be questionable with problems in the design and conduct of individual studies [40]. We found that 72 % of our meta-analyses reported having assessed the methodological quality or risk of bias but only 33 (18 %) considered the risk of confounding bias in their assessment. The Cochrane Collaboration has recognized the need to improve the assessment of risk of bias for NRSI and is currently developing a tool for this.

Finally, we found specific issues related to the inclusion of NRSI. In 69 % of the meta-analyses, the study design for each included study was unclear. The risk of bias may vary depending on the type of NRSI, with case–control studies generally considered as having a higher risk of bias than cohort studies. A description of the type of studies included in the meta-analysis is crucial. In addition, NRSI are prone to confounding: an imbalance in prognostic factors associated with the outcome of interest [24]. NRSI are expected to at least present adjusted estimates from multivariate analyses [3, 4]. Many of our meta-analyses (70 %) did not report or were unclear about whether the crude or adjusted estimates of NRSI were combined. Among the meta-analyses involving adjusted estimates, 33 % did not report the confounding factors adjusted for. This information was likely poorly reported in the individual studies, but then the reviewers should contact the authors for clarification or report it clearly in the meta-analysis.

## Conclusions

Some key methodological components of the systematic review process – search for grey literature, description of the type of NRSI included, assessment of risk of confounding bias and reporting of whether crude or adjusted estimates were combined—are not adequately reported in meta-analyses including NRSI. Attention should be paid to improving these elements in such meta-analyses to have an increased confidence in their results.

## Ethics

Not applicable. This article reports a meta-research study.

## Consent

Not needed. This study does not include human participants.

## Availability of supporting data

Data are available upon request for academic researchers.

## Appendices

### Appendix 1 Search strategy

Pubmed Search Equation

**Table:**

1.	“observational” [tiab]
2.	“cohort” [tiab]
3.	cohort studies [mh]
4.	“epidemiologic” [tiab]
5.	“epidemiological” [tiab]
6.	“systematic review” [tiab]
7.	“systematic reviews” [tiab]
8.	“meta-analysis” [tiab]
9.	“meta-analyses” [tiab]
10.	meta analysis [pt]
11.	“overview” [tiab]
12.	#1 OR #2 OR #3 OR #4 OR #5
13.	#6 OR #7 OR #8 OR #9 OR #10 OR #11
14.	#12 AND #13

*Date of search: 7 January 2014*

Search limits: Published between 1 January 2013 and 31 December 2013.

Citations retrieved: 3602.

### Appendix 2 List of included meta-analyses (*N* = 188)

Meta-analyses including observational studies and randomized controlled trials (*N* = 119)

**Table:**

Study ID	Reference of included citation
35	Abdelaal, E., S. V. Rao, et al. (2013). "Same-day discharge compared with overnight hospitalization after uncomplicated percutaneous coronary intervention: a systematic review and meta-analysis." JACC Cardiovasc Interv6(2): 99–112.
51	Abubakar, I., L. Pimpin, et al. (2013). "Systematic review and meta-analysis of the current evidence on the duration of protection by bacillus Calmette-Guerin vaccination against tuberculosis." Health Technol Assess17(37): 1–372, v-vi.
71	Agarwal, V., A. Briasoulis, et al. (2013). "Effects of renin-angiotensin system blockade on mortality and hospitalization in heart failure with preserved ejection fraction." Heart Fail Rev18(4): 429–437.
83	Ahmad, Z., R. Brooks, et al. (2013). "The effect of platelet-rich plasma on clinical outcomes in lateral epicondylitis." Arthroscopy29(11): 1851–1862.
89	Ahmed, M. and M. Douek (2013). "Intra-operative ultrasound versus wire-guided localization in the surgical management of non-palpable breast cancers: systematic review and meta-analysis." Breast Cancer Res Treat140(3): 435–446.
91	Ahmed, M. and M. Douek (2013). "Radioactive seed localisation (RSL) in the treatment of non-palpable breast cancers: systematic review and meta-analysis." Breast22(4): 383–388.
117	Alali, A. S., V. A. McCredie, et al. (2013). "Beta Blockers for Acute Traumatic Brain Injury: A Systematic Review and Meta-analysis." Neurocrit Care.
171	Almeida, C. C., M. R. Silveira, et al. (2013). "Safety of immunosuppressive drugs used as maintenance therapy in kidney transplantation: a systematic review and meta-analysis." Pharmaceuticals (Basel)6(10): 1170–1194.
253	Anglemyer, A., G. W. Rutherford, et al. (2013). "Antiretroviral therapy for prevention of HIV transmission in HIV-discordant couples." Cochrane Database Syst Rev4: CD009153.
265	Antoniou, G. A., N. Chalmers, et al. (2013). "A meta-analysis of endovascular versus surgical reconstruction of femoropopliteal arterial disease." J Vasc Surg57(1): 242–253.
271	Antoniou, G. A., G. S. Georgiadis, et al. (2013). "Endovascular repair for ruptured abdominal aortic aneurysm confers an early survival benefit over open repair." J Vasc Surg58(4): 1091–1105.
313	Arnaud, L., A. Mathian, et al. (2013). "Efficacy of aspirin for the primary prevention of thrombosis in patients with antiphospholipid antibodies: An international and collaborative meta-analysis." Autoimmun Rev.
329	Athappan, G., E. Patvardhan, et al. (2013). "Left main coronary artery stenosis: a meta-analysis of drug-eluting stents versus coronary artery bypass grafting." JACC Cardiovasc Interv6(12): 1219–1230.
385	Bagai, A., P. Thavendiranathan, et al. (2013). "Non-infarct-related artery revascularization during primary percutaneous coronary intervention for ST-segment elevation myocardial infarction: a systematic review and meta-analysis." Am Heart J166(4): 684–693 e681.
423	Bang, C. N., A. M. Greve, et al. (2013). "The preventive effect of statin therapy on new-onset and recurrent atrial fibrillation in patients not undergoing invasive cardiac interventions: a systematic review and meta-analysis." Int J Cardiol167(3): 624–630.
551	Beres, A. L. and R. Baird (2013). "An institutional analysis and systematic review with meta-analysis of pneumatic versus hydrostatic reduction for pediatric intussusception." Surgery154(2): 328–334.
637	Bittl, J. A., Y. He, et al. (2013). "Bayesian methods affirm the use of percutaneous coronary intervention to improve survival in patients with unprotected left main coronary artery disease." Circulation127(22): 2177–2185.
751	Brayton, K. M., V. G. Patel, et al. (2013). "Same-day discharge after percutaneous coronary intervention: a meta-analysis." J Am Coll Cardiol62(4): 275–285.
759	Breteler, J. K., J. S. Tam, et al. (2013). "Efficacy and effectiveness of seasonal and pandemic A (H1N1) 2009 influenza vaccines in low and middle income countries: a systematic review and meta-analysis." Vaccine31(45): 5168–5177.
885	Cao, C., S. C. Ang, et al. (2013). "Systematic review and meta-analysis of transcatheter aortic valve implantation versus surgical aortic valve replacement for severe aortic stenosis." Ann Cardiothorac Surg2(1): 10–23.
887	Cao, C., C. Manganas, et al. (2013). "Drug-eluting stents versus coronary artery bypass graft surgery in left main coronary artery disease: a meta-analysis of early outcomes from randomized and nonrandomized studies." J Thorac Cardiovasc Surg145(3): 738–747.
1009	Chant, C., A. Leung, et al. (2013). "Optimal dosing of antibiotics in critically ill patients using continuous/extended infusions: a systematic review and meta-analysis." Crit Care17(6): R279.
1023	Chatterjee, S., J. Wetterslev, et al. (2013). "Association of blood transfusion with increased mortality in myocardial infarction: a meta-analysis and diversity-adjusted study sequential analysis." JAMA Intern Med173(2): 132–139.
1069	Chen, Y., S. A. Nah, et al. (2013). "Transanal endorectal pull-through versus transabdominal approach for Hirschsprung's disease: a systematic review and meta-analysis." J Pediatr Surg48(3): 642–651.
1115	Chionh, C. Y., S. S. Soni, et al. (2013). "Use of peritoneal dialysis in AKI: a systematic review." Clin J Am Soc Nephrol8(10): 1649–1660.
1119	Chiumello, D., S. Coppola, et al. (2013). "Noninvasive ventilation in chest trauma: systematic review and meta-analysis." Intensive Care Med39(7): 1171–1180.
1131	Choi, Y. Y., J. M. Bae, et al. (2013). "Laparoscopic gastrectomy for advanced gastric cancer: Are the long-term results comparable with conventional open gastrectomy? A systematic review and meta-analysis." J Surg Oncol.
1147	Chowdhury, R., H. Khan, et al. (2013). "Adherence to cardiovascular therapy: a meta-analysis of prevalence and clinical consequences." Eur Heart J34(38): 2940–2948.
1375	De Luca, G., A. Schaffer, et al. (2013). "Comprehensive meta-analysis of radial vs femoral approach in primary angioplasty for STEMI." Int J Cardiol168(3): 2070–2081.
1451	Deng, Z., S. Zhang, et al. (2013). "Can statins reduce risk of lung cancer, especially among elderly people? A meta-analysis." Chin J Cancer Res25(6): 679–688.
1461	Deo, S. V., S. M. Dunlay, et al. (2013). "Dual anti-platelet therapy after coronary artery bypass grafting: is there any benefit? A systematic review and meta-analysis." J Card Surg28(2): 109–116.
1463	Deo, S. V., I. K. Shah, et al. (2013). "Myocardial revascularisation in renal dysfunction: a systematic review and meta-analysis." Heart Lung Circ22(10): 827–835.
1465	Deppe, A. C., O. J. Liakopoulos, et al. (2013). "Endoscopic vein harvesting for coronary artery bypass grafting: a systematic review with meta-analysis of 27,789 patients." J Surg Res180(1): 114–124.
1475	Desai, N., H. Bjornsson, et al. (2013). "Anatomic single- versus double-bundle ACL reconstruction: a meta-analysis." Knee Surg Sports Traumatol Arthrosc.
1525	Ding, J., G. Q. Liao, et al. (2013). "Medial versus lateral approach in laparoscopic colorectal resection: a systematic review and meta-analysis." World J Surg37(4): 863–872.
1757	Falagas, M. E., D. E. Karageorgopoulos, et al. (2013). "Continuous versus Conventional Infusion of Amphotericin B Deoxycholate: A Meta-Analysis." PLoS One8(10): e77075.
1779	Fan, X., K. Xu, et al. (2013). "Comparison of transperitoneal and retroperitoneal laparoscopic nephrectomy for renal cell carcinoma: a systematic review and meta-analysis." BJU Int111(4): 611–621.
1918	Ford, N., A. Calmy, et al. (2013). "Adverse events associated with nevirapine use in pregnancy: a systematic review and meta-analysis." AIDS27(7): 1135–1143.
1954	Franciosi, M., G. Lucisano, et al. (2013). "Metformin therapy and risk of cancer in patients with type 2 diabetes: systematic review." PLoS One8(8): e71583.
2190	Gomez Cuervo, C., O. Bisbal Pardo, et al. (2013). "Efficacy and safety of the use of heparin as thromboprophylaxis in patients with liver cirrhosis: a systematic review and meta-analysis." Thromb Res132(4): 414–419.
2351	Hagen, S. M., J. A. Lafranca, et al. (2013). "Laparoscopic versus open peritoneal dialysis catheter insertion: a meta-analysis." PLoS One8(2): e56351.
2389	Hanninen, M., N. Yeung-Lai-Wah, et al. (2013). "Cryoablation Versus RF Ablation for AVNRT: A Meta-Analysis and Systematic Review." J Cardiovasc Electrophysiol24(12): 1354–1360.
2423	Hartling, L., D. M. Dryden, et al. (2013). "Benefits and harms of treating gestational diabetes mellitus: a systematic review and meta-analysis for the U.S. Preventive Services Task Force and the National Institutes of Health Office of Medical Applications of Research." Ann Intern Med159(2): 123–129.
2439	Haut, E. R., L. J. Garcia, et al. (2013). "The Effectiveness of Prophylactic Inferior Vena Cava Filters in Trauma Patients: A Systematic Review and Meta-analysis." JAMA Surg.
2459	He, M. M., W. J. Wu, et al. (2013). "S-1-Based Chemotherapy versus Capecitabine-Based Chemotherapy as First-Line Treatment for Advanced Gastric Carcinoma: A Meta-Analysis." PLoS One8(12): e82798.
2463	He, S., Y. H. Tang, et al. (2013). "Pioglitazone prescription increases risk of bladder cancer in patients with type 2 diabetes: an updated meta-analysis." Tumour Biol.
2465	He, T., Y. Zhao, et al. (2013). "Pancreaticojejunostomy versus Pancreaticogastrostomy after Pancreaticoduodenectomy: A Systematic Review and Meta-Analysis." Dig Surg30(1): 56–69.
2569	Ho, E. T., G. Wong, et al. (2013). "Once-daily extended-release versus twice-daily standard-release tacrolimus in kidney transplant recipients: a systematic review." Transplantation95(9): 1120–1128.
2661	Hu, J., J. Qu, et al. (2013). "Allograft versus autograft for anterior cruciate ligament reconstruction: an up-to-date meta-analysis of prospective studies." Int Orthop37(2): 311–320.
2691	Huang, Y., Q. He, et al. (2013). "Antiarrhythmia drugs for cardiac arrest: a systemic review and meta-analysis." Crit Care17(4): R173.
2883	Jin, H. M., L. L. Guo, et al. (2013). "Effect of prolonged weekly hemodialysis on survival of maintenance hemodialysis patients: a meta-analysis of studies." Nephron Clin Pract123(3–4): 220–228.
2899	Johnson, L. N., I. E. Sasson, et al. (2013). "Does intracytoplasmic sperm injection improve the fertilization rate and decrease the total fertilization failure rate in couples with well-defined unexplained infertility? A systematic review and meta-analysis." Fertil Steril100(3): 704–711.
2901	Johnson, R. L., S. L. Kopp, et al. (2013). "Falls and major orthopaedic surgery with peripheral nerve blockade: a systematic review and meta-analysis." Br J Anaesth110(4): 518–528.
2975	Kalam, K. and T. H. Marwick (2013). "Role of cardioprotective therapy for prevention of cardiotoxicity with chemotherapy: a systematic review and meta-analysis." Eur J Cancer49(13): 2900–2909.
3129	Kim, H. S., J. E. Sardi, et al. (2013). "Efficacy of neoadjuvant chemotherapy in patients with FIGO stage IB1 to IIA cervical cancer: an international collaborative meta-analysis." Eur J Surg Oncol39(2): 115–124.
3286	Kuhn, E. W., O. J. Liakopoulos, et al. (2014). "Preoperative statin therapy in cardiac surgery: a meta-analysis of 90 000 patients." Eur J Cardiothorac Surg45(1): 17–26.
3294	Kulkarni, S., O. Wu, et al. (2013). "Centrally Inserted External Catheters and Totally Implantable Ports for the Delivery of Chemotherapy: A Systematic Review and Meta-Analysis of Device-Related Complications." Cardiovasc Intervent Radiol.
3516	Li, H., R. Pan, et al. (2013). "Clipping versus coiling for ruptured intracranial aneurysms: a systematic review and meta-analysis." Stroke44(1): 29–37.
3540	Li, Q., Z. Zhang, et al. (2013). "Drug-eluting stents or coronary artery bypass grafting for unprotected left main coronary artery disease: a meta-analysis of four randomized trials and seventeen observational studies." Trials14: 133.
3546	Li, S., Y. Liu, et al. (2013). "Association between non-steroidal anti-inflammatory drug use and melanoma risk: a meta-analysis of 13 studies." Cancer Causes Control24(8): 1505–1516.
3564	Li, Y., H. Zhang, et al. (2013). "Neuroendoscopic Surgery versus External Ventricular Drainage Alone or with Intraventricular Fibrinolysis for Intraventricular Hemorrhage Secondary to Spontaneous Supratentorial Hemorrhage: A Systematic Review and Meta-Analysis." PLoS One8(11): e80599.
3576	Liao, M., J. Huang, et al. (2013). "Transarterial chemoembolization in combination with local therapies for hepatocellular carcinoma: a meta-analysis." PLoS One8(7): e68453.
3658	Liu, J., L. P. Zhu, et al. (2013). "HMG-CoA reductase inhibitors (statins) and bone mineral density: a meta-analysis." Bone54(1): 151–156.
3694	Liu, X., Y. Wang, et al. (2013). "A systematic review with meta-analysis of posterior interbody fusion versus posterolateral fusion in lumbar spondylolisthesis." Eur Spine J.
3700	Liu, Y., Y. Lu, et al. (2013). "Association between non-steroidal anti-inflammatory drug use and brain tumour risk: a meta-analysis." Br J Clin Pharmacol.
4083	Mehta, V., T. S. Vasu, et al. (2013). "Obstructive sleep apnea and oxygen therapy: a systematic review of the literature and meta-analysis." J Clin Sleep Med9(3): 271–279.
4111	Mesgarpour, B., B. H. Heidinger, et al. (2013). "Safety of off-label erythropoiesis stimulating agents in critically ill patients: a meta-analysis." Intensive Care Med39(11): 1896–1908.
4125	Meybohm, P., E. Herrmann, et al. (2013). "Aprotinin may increase mortality in low and intermediate risk but not in high risk cardiac surgical patients compared to tranexamic acid and epsilon-aminocaproic acid -- a meta-analysis of randomised and observational trials of over 30.000 patients." PLoS One8(3): e58009.
4223	Moran, P. S., M. O'Neill, et al. (2013). "Robot-assisted radical prostatectomy compared with open and laparoscopic approaches: a systematic review and meta-analysis." Int J Urol20(3): 312–321.
4263	Moyses, H. E., M. J. Johnson, et al. (2013). "Early parenteral nutrition and growth outcomes in preterm infants: a systematic review and meta-analysis." Am J Clin Nutr97(4): 816–826.
4297	Murji, A., V. I. Patel, et al. (2013). "Single-incision laparoscopy in gynecologic surgery: a systematic review and meta-analysis." Obstet Gynecol121(4): 819–828.
4359	Navarese, E. P., P. A. Gurbel, et al. (2013). "Optimal timing of coronary invasive strategy in non-ST-segment elevation acute coronary syndromes: a systematic review and meta-analysis." Ann Intern Med158(4): 261–270.
4409	Ni Chroinin, D., K. Asplund, et al. (2013). "Statin therapy and outcome after ischemic stroke: systematic review and meta-analysis of observational studies and randomized trials." Stroke44(2): 448–456.
4411	Ni, X., J. Ma, et al. (2013). "Meta-analysis on the association between non-steroidal anti-inflammatory drug use and ovarian cancer." Br J Clin Pharmacol75(1): 26–35.
4681	Park, H. J., E. J. Nam, et al. (2013). "The benefit of adjuvant chemotherapy combined with postoperative radiotherapy for endometrial cancer: a meta-analysis." Eur J Obstet Gynecol Reprod Biol170(1): 39–44.
4807	Petrelli, F., K. Borgonovo, et al. (2013). "FOLFIRI-bevacizumab as first-line chemotherapy in 3500 patients with advanced colorectal cancer: a pooled analysis of 29 published trials." Clin Colorectal Cancer12(3): 145–151.
4841	Phung, O. J., E. Schwartzman, et al. (2013). "Sulphonylureas and risk of cardiovascular disease: systematic review and meta-analysis." Diabet Med30(10): 1160–1171.
4853	Pillay, P., N. Ford, et al. (2013). "Outcomes for efavirenz versus nevirapine-containing regimens for treatment of HIV-1 infection: a systematic review and meta-analysis." PLoS One8(7): e68995.
4871	Pisanu, A., G. Porceddu, et al. (2013). "Meta-analysis of studies comparing single-incision laparoscopic appendectomy and conventional multiport laparoscopic appendectomy." J Surg Res183(2): e49-59.
5017	Rabie, R., K. Mumtaz, et al. (2013). "Efficacy of antiviral therapy for hepatitis C after liver transplantation with cyclosporine and tacrolimus: a systematic review and meta-analysis." Liver Transpl19(1): 36–48.
5143	Richardson, K., M. Schoen, et al. (2013). "Statins and cognitive function: a systematic review." Ann Intern Med159(10): 688–697.
5203	Romeo, F., M. C. Acconcia, et al. (2013). "Lack of intra-aortic balloon pump effectiveness in high-risk percutaneous coronary interventions without cardiogenic shock: a comprehensive meta-analysis of randomised trials and observational studies." Int J Cardiol167(5): 1783–1793.
5365	Santangeli, P., R. Proietti, et al. (2013). "Cryoablation versus radiofrequency ablation of atrioventricular nodal reentrant tachycardia." J Interv Card Electrophysiol.
5428	Schneider, A. G., R. Bellomo, et al. (2013). "Choice of renal replacement therapy modality and dialysis dependence after acute kidney injury: a systematic review and meta-analysis." Intensive Care Med39(6): 987–997.
5464	Schweizer, M., E. Perencevich, et al. (2013). "Effectiveness of a bundled intervention of decolonization and prophylaxis to decrease Gram positive surgical site infections after cardiac or orthopedic surgery: systematic review and meta-analysis." BMJ346: f2743.
5666	Singh, S., H. Singh, et al. (2013). "Antidiabetic Medications and the Risk of Colorectal Cancer in Patients with Diabetes Mellitus: A Systematic Review and Meta-analysis." Cancer Epidemiol Biomarkers Prev22(12): 2258–2268.
5670	Singh, S., P. P. Singh, et al. (2013). "Anti-diabetic medications and risk of pancreatic cancer in patients with diabetes mellitus: a systematic review and meta-analysis." Am J Gastroenterol108(4): 510–519; quiz 520.
5672	Singh, S., P. P. Singh, et al. (2013). "Anti-diabetic medications and the risk of hepatocellular cancer: a systematic review and meta-analysis." Am J Gastroenterol108(6): 881–891; quiz 892.
5736	Soares-Weiser, K., L. Bechard-Evans, et al. (2013). "Time to all-cause treatment discontinuation of olanzapine compared to other antipsychotics in the treatment of schizophrenia: a systematic review and meta-analysis." Eur Neuropsychopharmacol23(2): 118–125.
5926	Sundaresh, V., J. P. Brito, et al. (2013). "Comparative effectiveness of therapies for Graves' hyperthyroidism: a systematic review and network meta-analysis." J Clin Endocrinol Metab98(9): 3671–3677.
5962	Takagi, H., M. Niwa, et al. (2013). "A meta-analysis of transcatheter aortic valve implantation versus surgical aortic valve replacement." Ann Thorac Surg96(2): 513–519.
5988	Tan, M., X. Song, et al. (2013). "Statins and the risk of lung cancer: a meta-analysis." PLoS One8(2): e57349.
6066	Thakkar, B., K. N. Aronis, et al. (2013). "Metformin and sulfonylureas in relation to cancer risk in type II diabetes patients: a meta-analysis using primary data of published studies." Metabolism62(7): 922–934.
6080	Thiele, M., L. L. Gluud, et al. (2013). "Antiviral therapy for prevention of hepatocellular carcinoma and mortality in chronic hepatitis B: systematic review and meta-analysis." BMJ Open3(8).
6204	Tseng, S. H., H. C. Chen, et al. (2013). "Systematic review and meta-analysis of the effect of equine assisted activities and therapies on gross motor outcome in children with cerebral palsy." Disabil Rehabil35(2): 89–99.
6224	Turner, R. M., C. S. Kwok, et al. (2013). "Thiazolidinediones and associated risk of Bladder Cancer: a Systematic Review and Meta-analysis." Br J Clin Pharmacol.
6234	Twine, C. P., A. K. Humphreys, et al. (2013). "Systematic review and meta-analysis of the retroperitoneal versus the transperitoneal approach to the abdominal aorta." Eur J Vasc Endovasc Surg46(1): 36–47.
6237	Ueda, T., Y. Suzukamo, et al. (2013). "Effects of music therapy on behavioral and psychological symptoms of dementia: a systematic review and meta-analysis." Ageing Res Rev12(2): 628–641.
6265	Valkhoff, V. E., M. Sturkenboom, et al. (2013). "Low-dose acetylsalicylic acid use and the risk of upper gastrointestinal bleeding: A meta-analysis of randomized clinical trials and observational studies." Can J Gastroenterol27(3): 159–167.
6383	Vercellini, P., D. E. M. S, et al. (2013). "Long-term adjuvant therapy for the prevention of postoperative endometrioma recurrence: a systematic review and meta-analysis." Acta Obstet Gynecol Scand92(1): 8–16.
6461	Vyas, A., M. Schweizer, et al. (2013). "Meta-Analysis of Same Versus Different Stent for Drug-Eluting Stent Restenosis." Am J Cardiol.
6474	Wan, B., M. Rahnavardi, et al. (2013). "A meta-analysis of MitraClip system versus surgery for treatment of severe mitral regurgitation." Ann Cardiothorac Surg2(6): 683–692.
6512	Wang, J., C. Li, et al. (2013). "Statin use and risk of lung cancer: a meta-analysis of observational studies and randomized controlled trials." PLoS One8(10): e77950.
6518	Wang, J., X. Xie, et al. (2013). "Locoregional and distant recurrences after breast conserving therapy in patients with triple-negative breast cancer: A meta-analysis." Surg Oncol22(4): 247–255.
6579	Wang, Z., M. Nabhan, et al. (2013). "Charged particle radiation therapy for uveal melanoma: a systematic review and meta-analysis." Int J Radiat Oncol Biol Phys86(1): 18–26.
6585	Wang, Z. J., K. J. Harjai, et al. (2013). "Drug-eluting stents versus bare-metal stents in patients with decreased GFR: a meta-analysis." Am J Kidney Dis62(4): 711–721.
6663	Wilasrusmee, C., M. Marjareonrungrung, et al. (2013). "Maggot therapy for chronic ulcer: A retrospective cohort and a meta-analysis." Asian J Surg.
6683	Williams, N. H., R. Lewis, et al. (2013). "A systematic review and meta-analysis of biological treatments targeting tumour necrosis factor alpha for sciatica." Eur Spine J22(9): 1921–1935.
6709	Wolfrum, M., G. M. Froehlich, et al. (2013). "Stroke prevention by percutaneous closure of patent foramen ovale: a systematic review and meta-analysis." Heart.
6717	Wong, W. B., V. W. Lin, et al. (2013). "Statins in the prevention of dementia and Alzheimer's disease: a meta-analysis of observational studies and an assessment of confounding." Pharmacoepidemiol Drug Saf22(4): 345–358.
6825	Xiong, J. J., K. Altaf, et al. (2013). "Roux-en-Y versus Billroth I reconstruction after distal gastrectomy for gastric cancer: a meta-analysis." World J Gastroenterol19(7): 1124–1134.
6829	Xu, C. and J. Zhao (2013). "A meta-analysis comparing meniscal repair with meniscectomy in the treatment of meniscal tears: the more meniscus, the better outcome?" Knee Surg Sports Traumatol Arthrosc.
6872	Yan, S., D. Xu, et al. (2013). "Combination of radiofrequency ablation with transarterial chemoembolization for hepatocellular carcinoma: a meta-analysis." Dig Dis Sci58(7): 2107–2113.
7000	Zampieri, F. G., P. V. Mendes, et al. (2013). "Extracorporeal membrane oxygenation for severe respiratory failure in adult patients: a systematic review and meta-analysis of current evidence." J Crit Care28(6): 998–1005.
7055	Zhang, X. L., J. Geng, et al. (2013). "Statin use and risk of bladder cancer: a meta-analysis." Cancer Causes Control24(4): 769–776.
7057	Zhang, X. L., M. Liu, et al. (2013). "Statin use and risk of kidney cancer: a meta-analysis of observational studies and randomized trials." Br J Clin Pharmacol.
7118	Zhong, J. H., X. S. Mo, et al. (2013). "Postoperative use of the chemopreventive vitamin K2 analog in patients with hepatocellular carcinoma." PLoS One8(3): e58082.
7150	Zhu, W., Y. Wu, et al. (2013). "Aspirin use and risk of age-related macular degeneration: a meta-analysis." PLoS One8(3): e58821.
7162	Zimarino, M., A. Corazzini, et al. (2013). "Late thrombosis after double versus single drug-eluting stent in the treatment of coronary bifurcations: a meta-analysis of randomized and observational Studies." JACC Cardiovasc Interv6(7): 687–695.

Meta-analyses including only observational studies (*N* = 69)

**Table:**

Study ID	Reference of included citation
93	Ahmed, S., R. K. Shahid, et al. (2013). "Should noncurative resection of the primary tumour be performed in patients with stage iv colorectal cancer? A systematic review and meta-analysis." Curr Oncol20(5): e420-441.
113	Al Rawahi, T., A. D. Lopes, et al. (2013). "Surgical cytoreduction for recurrent epithelial ovarian cancer." Cochrane Database Syst Rev2: CD008765.
151	Alhazmi, A., E. Stojanovski, et al. (2013). "The association between dietary patterns and type 2 diabetes: a systematic review and meta-analysis of cohort studies." J Hum Nutr Diet.
227	Andrade, J. G., M. W. Deyell, et al. (2013). "Risk of bleeding on triple antithrombotic therapy after percutaneous coronary intervention/stenting: a systematic review and meta-analysis." Can J Cardiol29(2): 204–212.
269	Antoniou, G. A., G. S. Georgiadis, et al. (2013). "A meta-analysis of outcomes of endovascular abdominal aortic aneurysm repair in patients with hostile and friendly neck anatomy." J Vasc Surg57(2): 527–538.
375	Bacchetti, S., S. Bertozzi, et al. (2013). "Surgical treatment and survival in patients with liver metastases from neuroendocrine tumors: a meta-analysis of observational studies." Int J Hepatol2013: 235040.
433	Bao, F., P. Ye, et al. (2013). "Segmentectomy or lobectomy for early stage lung cancer: a meta-analysis." Eur J Cardiothorac Surg.
459	Bartels, S. A., T. J. Gardenbroek, et al. (2013). "Systematic review and meta-analysis of laparoscopic versus open colectomy with end ileostomy for non-toxic colitis." Br J Surg100(6): 726–733.
481	Bavinger, C., E. Bendavid, et al. (2013). "Risk of cardiovascular disease from antiretroviral therapy for HIV: a systematic review." PLoS One8(3): e59551.
491	Beales, I. L., A. Hensley, et al. (2013). "Reduced esophageal cancer incidence in statin users, particularly with cyclo-oxygenase inhibition." World J Gastrointest Pharmacol Ther4(3): 69–79.
525	Belkhair, S. and G. Pickett (2013). "One versus double burr holes for treating chronic subdural hematoma meta-analysis." Can J Neurol Sci40(1): 56–60.
559	Berhan, Y. and A. Berhan (2013). "A meta-analysis of reverse breech extraction to deliver a deeply impacted head during cesarean delivery." Int J Gynaecol Obstet.
627	Billioud, V., A. C. Ford, et al. (2013). "Preoperative use of anti-TNF therapy and postoperative complications in inflammatory bowel diseases: a meta-analysis." J Crohns Colitis7(11): 853–867.
791	Brown, K. A., N. Khanafer, et al. (2013). "Meta-analysis of antibiotics and the risk of community-associated Clostridium difficile infection." Antimicrob Agents Chemother57(5): 2326–2332.
916	Carnuccio, P., J. Jimeno, et al. (2013). "Laparoscopic right colectomy: a systematic review and meta-analysis of observational studies comparing two types of anastomosis." Tech Coloproctol.
990	Chang, K. H., J. P. Burke, et al. (2013). "Infliximab versus cyclosporine as rescue therapy in acute severe steroid-refractory ulcerative colitis: a systematic review and meta-analysis." Int J Colorectal Dis28(3): 287–293.
1025	Chatu, S., V. Subramanian, et al. (2013). "The Role of Thiopurines in Reducing the Need for Surgical Resection in Crohn's Disease: A Systematic Review and Meta-Analysis." Am J Gastroenterol.
1063	Chen, W. K. and C. H. Miao (2013). "The effect of anesthetic technique on survival in human cancers: a meta-analysis of retrospective and prospective studies." PLoS One8(2): e56540.
1065	Chen, Y., J. J. Guan, et al. (2013). "Outcome of Cervicocranial Artery Dissection with Different Treatments: A Systematic Review and Meta-analysis." J Stroke Cerebrovasc Dis.
1161	Chu, H., L. Zhao, et al. (2013). "Sulbactam-based therapy for Acinetobacter baumannii infection: a systematic review and meta-analysis." Braz J Infect Dis17(4): 389–394.
1367	de Cuba, E. M., R. Kwakman, et al. (2013). "Cytoreductive surgery and HIPEC for peritoneal metastases combined with curative treatment of colorectal liver metastases: Systematic review of all literature and meta-analysis of observational studies." Cancer Treat Rev39(4): 321–327.
1399	De Vecchis, R., C. Esposito, et al. (2013). "Cabergoline use and risk of fibrosis and insufficiency of cardiac valves : Meta-analysis of observational studies." Herz38(8): 868–880.
1621	Duranton, F., M. E. Rodriguez-Ortiz, et al. (2013). "Vitamin D treatment and mortality in chronic kidney disease: a systematic review and meta-analysis." Am J Nephrol37(3): 239–248.
1675	El-Hussuna, A., A. Krag, et al. (2013). "The effect of anti-tumor necrosis factor alpha agents on postoperative anastomotic complications in Crohn's disease: a systematic review." Dis Colon Rectum56(12): 1423–1433.
1741	Eurich, D. T., D. L. Weir, et al. (2013). "Comparative safety and effectiveness of metformin in patients with diabetes mellitus and heart failure: systematic review of observational studies involving 34,000 patients." Circ Heart Fail6(3): 395–402.
1932	Forst, T., M. Hanefeld, et al. (2013). "Association of sulphonylurea treatment with all-cause and cardiovascular mortality: a systematic review and meta-analysis of observational studies." Diab Vasc Dis Res10(4): 302–314.
1980	Fu, D., G. Li, et al. (2013). "Comparison of clinical outcome between simultaneous-bilateral and staged-bilateral total knee arthroplasty: a systematic review of retrospective studies." J Arthroplasty28(7): 1141–1147.
2044	Gandhi, S., N. Narula, et al. (2013). "Meta-analysis: colonoscopic post-polypectomy bleeding in patients on continued clopidogrel therapy." Aliment Pharmacol Ther37(10): 947–952.
2112	Gedmintas, L., D. H. Solomon, et al. (2013). "Bisphosphonates and risk of subtrochanteric, femoral shaft, and atypical femur fracture: a systematic review and meta-analysis." J Bone Miner Res28(8): 1729–1737.
2196	Gong, J., L. Zhu, et al. (2013). "Use of thiopurines and risk of colorectal neoplasia in patients with inflammatory bowel diseases: a meta-analysis." PLoS One8(11): e81487.
2343	Habib, A. G. and D. A. Warrell (2013). "Antivenom therapy of carpet viper (Echis ocellatus) envenoming: effectiveness and strategies for delivery in West Africa." Toxicon69: 82–89.
2441	Haverkamp, L., T. J. Weijs, et al. (2013). "Laparoscopic total gastrectomy versus open total gastrectomy for cancer: a systematic review and meta-analysis." Surg Endosc27(5): 1509–1520.
2733	Hyun, M. H., C. H. Lee, et al. (2013). "Systematic review and meta-analysis of robotic surgery compared with conventional laparoscopic and open resections for gastric carcinoma." Br J Surg100(12): 1566–1578.
2795	Jackson, C., A. Mann, et al. (2013). "Effectiveness of Haemophilus influenzae type b vaccines administered according to various schedules: systematic review and meta-analysis of observational data." Pediatr Infect Dis J32(11): 1261–1269.
2825	Jamjoom, A. A. and A. B. Jamjoom (2013). "Safety and efficacy of early pharmacological thromboprophylaxis in traumatic brain injury: systematic review and meta-analysis." J Neurotrauma30(7): 503–511.
3030	Karthikeyan, G., N. B. Senguttuvan, et al. (2013). "Urgent surgery compared with fibrinolytic therapy for the treatment of left-sided prosthetic heart valve thrombosis: a systematic review and meta-analysis of observational studies." Eur Heart J34(21): 1557–1566.
3097	Khan, A. R., M. Riaz, et al. (2013). "The role of statins in prevention and treatment of community acquired pneumonia: a systematic review and meta-analysis." PLoS One8(1): e52929.
3496	Li, B. H., X. Ding, et al. (2013). "Meta-analysis of clinical outcomes of intravenous recombinant tissue plasminogen activator for acute ischemic stroke: within 3 h versus 3–4.5 h." Curr Med Res Opin29(9): 1105–1114.
3692	Liu, X., S. Min, et al. (2013). "Anterior corpectomy versus posterior laminoplasty for multilevel cervical myelopathy: a systematic review and meta-analysis." Eur Spine J.
4032	Mazza, M., F. D'Ascenzo, et al. (2013). "Drugs for attention deficit-hyperactivity disorder do not increase the mid-term risk of sudden death in children: a meta-analysis of observational studies." Int J Cardiol168(4): 4320–4321.
4335	Nakamura, M. and H. Nakashima (2013). "Laparoscopic distal pancreatectomy and pancreatoduodenectomy: is it worthwhile? A meta-analysis of laparoscopic pancreatectomy." J Hepatobiliary Pancreat Sci20(4): 421–428.
4375	Nderitu, P., L. Doos, et al. (2013). "Non-steroidal anti-inflammatory drugs and chronic kidney disease progression: a systematic review." Fam Pract30(3): 247–255.
4528	O'Neill, M., P. S. Moran, et al. (2013). "Robot-assisted hysterectomy compared to open and laparoscopic approaches: systematic review and meta-analysis." Arch Gynecol Obstet287(5): 907–918.
4679	Park, H. J., D. W. Kim, et al. (2013). "Staging laparoscopy for the management of early-stage ovarian cancer: a metaanalysis." Am J Obstet Gynecol209(1): 58 e51-58.
4731	Pazionis, T. J., H. Alradwan, et al. (2013). "A Systematic Review and Meta-Analysis of En-Bloc vs Intralesional Resection for Giant Cell Tumor of Bone of the Distal Radius." Open Orthop J7: 103–108.
5377	Sarikaya, H., B. R. da Costa, et al. (2013). "Antiplatelets versus anticoagulants for the treatment of cervical artery dissection: Bayesian meta-analysis." PLoS One8(9): e72697.
5496	Sepehripour, A. H., S. Saso, et al. (2013). "Does off-pump coronary revascularization reduce mortality in re-operative coronary artery surgery? A meta-analysis of observational studies." Perfusion28(4): 340–349.
5528	Shan, L., P. Hao, et al. (2013). "Benefits of early tracheotomy: a meta-analysis based on 6 observational studies." Respir Care58(11): 1856–1862.
5548	Sharma, V., S. V. Deo, et al. (2014). "Meta-analysis of staged versus combined carotid endarterectomy and coronary artery bypass grafting." Ann Thorac Surg97(1): 102–109.
5744	Soeters, H. M., S. Napravnik, et al. (2013). "The effect of tuberculosis treatment on virologic and CD4+ cell count response to combination antiretroviral therapy: a systematic review." AIDS.
5746	Soeters, H. M., C. Poole, et al. (2013). "The effect of tuberculosis treatment at combination antiretroviral therapy initiation on subsequent mortality: a systematic review and meta-analysis." PLoS One8(10): e78073.
5840	Steinberg, B. A., V. Hasselblad, et al. (2013). "Dabigatran for periprocedural anticoagulation following radiofrequency ablation for atrial fibrillation: a meta-analysis of observational studies." J Interv Card Electrophysiol37(3): 213–221.
5882	Su, A. P., N. W. Ke, et al. (2014). "Is laparoscopic approach for pancreatic insulinomas safe? Results of a systematic review and meta-analysis." J Surg Res186(1): 126–134.
5958	Taioli, E., D. S. Lee, et al. (2013). "Long-term survival in video-assisted thoracoscopic lobectomy vs open lobectomy in lung-cancer patients: a meta-analysis." Eur J Cardiothorac Surg44(4): 591–597.
6040	Tee, M. C., Y. Cao, et al. (2013). "Effect of bariatric surgery on oncologic outcomes: a systematic review and meta-analysis." Surg Endosc27(12): 4449–4456.
6092	Thosani, N., S. N. Thosani, et al. (2013). "Reduced risk of colorectal cancer with use of oral bisphosphonates: a systematic review and meta-analysis." J Clin Oncol31(5): 623–630.
6526	Wang, L., Z. Wu, et al. (2013). "Laparoendoscopic single-site adrenalectomy versus conventional laparoscopic surgery: a systematic review and meta-analysis of observational studies." J Endourol27(6): 743–750.
6621	Weiss, A. J., S. Zhao, et al. (2013). "A meta-analysis comparing bilateral internal mammary artery with left internal mammary artery for coronary artery bypass grafting." Ann Cardiothorac Surg2(4): 390–400.
6779	Wu, X. S., P. Dong, et al. (2013). "Combined portal vein resection for hilar cholangiocarcinoma: a meta-analysis of comparative studies." J Gastrointest Surg17(6): 1107–1115.
6843	Xu, S. B., Y. P. Zhu, et al. (2013). "Patients get more long-term benefit from central pancreatectomy than distal resection: a meta-analysis." Eur J Surg Oncol39(6): 567–574.
6866	Yakoob, M. Y., B. T. Bateman, et al. (2013). "The risk of congenital malformations associated with exposure to beta-blockers early in pregnancy: a meta-analysis." Hypertension62(2): 375–381.
6912	Ye, X., J. Fu, et al. (2013). "Dose-risk and duration-risk relationships between aspirin and colorectal cancer: a meta-analysis of published cohort studies." PLoS One8(2): e57578.
6944	Yoon, J. M., E. G. Cho, et al. (2013). "Antidepressant use and diabetes mellitus risk: a meta-analysis." Korean J Fam Med34(4): 228–240.
7018	Zhang, H., C. Gao, et al. (2013). "Metformin and reduced risk of hepatocellular carcinoma in diabetic patients: a meta-analysis." Scand J Gastroenterol48(1): 78–87.
7020	Zhang, H., D. Jiang, et al. (2013). "Use of nonsteroidal anti-inflammatory drugs and bladder cancer risk: a meta-analysis of epidemiologic studies." PLoS One8(7): e70008.
7096	Zheng, H., S. Xue, et al. (2013). "Meta-analysis of clinical studies comparing coronary artery bypass grafting with percutaneous coronary intervention in patients with end-stage renal disease." Eur J Cardiothorac Surg43(3): 459–467.
7104	Zheng, Z., W. Liang, et al. (2013). "Liver Transplantation Versus Liver Resection in the Treatment of Hepatocellular Carcinoma: A Meta-Analysis of Observational Studies." Transplantation.
7108	Zheng, Z., H. Shi, et al. (2013). "Vitamin D supplementation and mortality risk in chronic kidney disease: a meta-analysis of 20 observational studies." BMC Nephrol14: 199.
7130	Zhou, X., J. L. Du, et al. (2013). "Statin therapy is beneficial for the prevention of atrial fibrillation in patients with coronary artery disease: a meta-analysis." Eur J Pharmacol707(1–3): 104–111.

### Appendix 3: List of excluded meta-analyses (*N* = 153)

**Table:**

Reason for exclusion:	
Not a therapeutic evaluation (*n* = 77)	
Study ID	Reference of included citation
177	Almenawer, S. A., I. Bogza, et al. (2013). "The value of scheduled repeat cranial computed tomography after mild head injury: single-center series and meta-analysis." Neurosurgery72(1): 56–62; discussion 63–54.
335	Atlantis, E., P. Fahey, et al. (2013). "Endogenous testosterone level and testosterone supplementation therapy in chronic obstructive pulmonary disease (COPD): a systematic review and meta-analysis." BMJ Open3(8).
2050	Gans, I., K. D. Baldwin, et al. (2013). "Treatment and Management Outcomes of Tibial Eminence Fractures in Pediatric Patients: A Systematic Review." Am J Sports Med.
2062	Garces, S., J. Demengeot, et al. (2013). "The immunogenicity of anti-TNF therapy in immune-mediated inflammatory diseases: a systematic review of the literature with a meta-analysis." Ann Rheum Dis72(12): 1947–1955.
5938	Suthar, A. B., D. Hoos, et al. (2013). "Integrating antiretroviral therapy into antenatal care and maternal and child health settings: a systematic review and meta-analysis." Bull World Health Organ91(1): 46–56.
7112	Zhi, X., X. Zhou, et al. (2013). "Practical role of mutation analysis for imatinib treatment in patients with advanced gastrointestinal stromal tumors: a meta-analysis." PLoS One8(11): e79275.
475	Bauer, S. R., S. E. Hankinson, et al. (2013). "Plasma vitamin D levels, menopause, and risk of breast cancer: dose–response meta-analysis of prospective studies." Medicine (Baltimore)92(3): 123–131.
591	Bewtra, M., L. M. Kaiser, et al. (2013). "Crohn's disease and ulcerative colitis are associated with elevated standardized mortality ratios: a meta-analysis." Inflamm Bowel Dis19(3): 599–613.
597	Bhangu, A., D. Nepogodiev, et al. (2013). "Meta-analysis of plasma to red blood cell ratios and mortality in massive blood transfusions for trauma." Injury44(12): 1693–1699.
675	Bonifazi, M., I. Tramacere, et al. (2013). "Systemic sclerosis (scleroderma) and cancer risk: systematic review and meta-analysis of observational studies." Rheumatology (Oxford)52(1): 143–154.
701	Bosanquet, D. C., D. A. Harris, et al. (2013). "Systematic review and meta-analysis of intraoperative peritoneal lavage for colorectal cancer staging." Br J Surg100(7): 853–862.
717	Bougma, K., F. E. Aboud, et al. (2013). "Iodine and mental development of children 5 years old and under: a systematic review and meta-analysis." Nutrients5(4): 1384–1416.
803	Bryant, A. R., S. B. Wilton, et al. (2013). "Association between QRS duration and outcome with cardiac resynchronization therapy: a systematic review and meta-analysis." J Electrocardiol46(2): 147–155.
1017	Charidimou, A., P. Kakar, et al. (2013). "Cerebral microbleeds and recurrent stroke risk: systematic review and meta-analysis of prospective ischemic stroke and transient ischemic attack cohorts." Stroke44(4): 995–1001.
1349	Davies, A., K. P. Singh, et al. (2013). "Treatment outcomes of treatment-naive Hepatitis C patients co-infected with HIV: a systematic review and meta-analysis of observational cohorts." PLoS One8(2): e55373.
1996	Fumery, M., C. Xiaocang, et al. (2013). "Thromboembolic events and cardiovascular mortality in inflammatory bowel diseases: A meta-analysis of observational studies." J Crohns Colitis.
2066	Garcia, S., Y. Sandoval, et al. (2013). "Outcomes after complete versus incomplete revascularization of patients with multivessel coronary artery disease: a meta-analysis of 89,883 patients enrolled in randomized clinical trials and observational studies." J Am Coll Cardiol62(16): 1421–1431.
2144	Gielen, C., O. Dekkers, et al. (2013). "The effects of pre- and postoperative fibrinogen levels on blood loss after cardiac surgery: a systematic review and meta-analysis." Interact Cardiovasc Thorac Surg.
2689	Huang, Y., R. Chen, et al. (2013). "Association between point-of-care CRP testing and antibiotic prescribing in respiratory tract infections: a systematic review and meta-analysis of primary care studies." Br J Gen Pract63(616): 787–794.
2823	James, M. T., S. M. Samuel, et al. (2013). "Contrast-induced acute kidney injury and risk of adverse clinical outcomes after coronary angiography: a systematic review and meta-analysis." Circ Cardiovasc Interv6(1): 37–43.
3014	Kaptoge, S., S. R. Seshasai, et al. (2013). "Inflammatory cytokines and risk of coronary heart disease: new prospective study and updated meta-analysis." Eur Heart J.
3476	Lemos, E. V., F. P. de la Hoz, et al. (2013). "Carbapenem resistance and mortality in patients with Acinetobacter baumannii infection: systematic review and meta-analysis." Clin Microbiol Infect.
317	Arregui, M., B. Buijsse, et al. (2014). "Adiponectin and Risk of Stroke: Prospective Study and Meta-analysis." Stroke45(1): 10–17.
367	Baandrup, L., M. T. Faber, et al. (2013). "Nonsteroidal anti-inflammatory drugs and risk of ovarian cancer: systematic review and meta-analysis of observational studies." Acta Obstet Gynecol Scand92(3): 245–255.
609	Bhattacharjee, S., R. Bhattacharya, et al. (2013). "Antidepressant use and new-onset diabetes: a systematic review and meta-analysis." Diabetes Metab Res Rev29(4): 273–284.
641	Blackadar, C. B. (2013). "Systematic review of hepatocellular carcinoma mortality rates among hepatitis B virus-infected renal transplant recipients, with supplemental analyses of liver failure and all-cause mortality." Int J Infect Dis17(1): e24-36.
679	Bonovas, S., G. Nikolopoulos, et al. (2013). "Bisphosphonate use and risk of colorectal cancer: a systematic review and meta-analysis." Br J Clin Pharmacol76(3): 329–337.
707	Bosetti, C., V. Rosato, et al. (2013). "Cancer risk for patients using thiazolidinediones for type 2 diabetes: a meta-analysis." Oncologist18(2): 148–156.
771	Brinton, L. A. and A. S. Felix (2013). "Menopausal hormone therapy and risk of endometrial cancer." J Steroid Biochem Mol Biol.
1071	Chen, Y. B., Q. Chen, et al. (2013). "Insulin therapy and risk of prostate cancer: a systematic review and meta-analysis of observational studies." PLoS One8(11): e81594.
1143	Choueiri, T. K., Y. Je, et al. (2013). "Analgesic use and the risk of kidney cancer: A meta-analysis of epidemiologic studies." Int J Cancer.
2415	Hargreave, M., A. Jensen, et al. (2013). "Fertility treatment and childhood cancer risk: a systematic meta-analysis." Fertil Steril100(1): 150–161.
2443	Havrilesky, L. J., P. G. Moorman, et al. (2013). "Oral contraceptive pills as primary prevention for ovarian cancer: a systematic review and meta-analysis." Obstet Gynecol122(1): 139–147.
3024	Karlstad, O., J. Starup-Linde, et al. (2013). "Use of insulin and insulin analogs and risk of cancer - systematic review and meta-analysis of observational studies." Curr Drug Saf8(5): 333–348.
3464	Lee, S. H., R. C. Chan, et al. (2013). "Use of bisphosphonates and the risk of osteonecrosis among cancer patients: a systemic review and meta-analysis of the observational studies." Support Care Cancer.
3466	Lee, S. H., S. S. Chang, et al. (2013). "Risk of osteonecrosis in patients taking bisphosphonates for prevention of osteoporosis: a systematic review and meta-analysis." Osteoporos Int.
3933	Maneiro, J. R., E. Salgado, et al. (2013). "Immunogenicity of monoclonal antibodies against tumor necrosis factor used in chronic immune-mediated Inflammatory conditions: systematic review and meta-analysis." JAMA Intern Med173(15): 1416–1428.
4345	Namazy, J. A., V. E. Murphy, et al. (2013). "Effects of asthma severity, exacerbations and oral corticosteroids on perinatal outcomes." Eur Respir J41(5): 1082–1090.
4441	Noel, S. E., A. C. Stoneham, et al. (2013). "Consumption of omega-3 fatty acids and the risk of skin cancers: A systematic review and meta-analysis." Int J Cancer.
4695	Parsaik, A. K., B. Singh, et al. (2013). "Statins use and risk of depression: A systematic review and meta-analysis." J Affect Disord.
4923	Pradelli, D., D. Soranna, et al. (2013). "Statins and primary liver cancer: a meta-analysis of observational studies." Eur J Cancer Prev22(3): 229–234.
4971	Qi, Z. Y., C. Shao, et al. (2013). "Reproductive and exogenous hormone factors in relation to risk of meningioma in women: a meta-analysis." PLoS One8(12): e83261.
4975	Qin, J., T. Yang, et al. (2013). "Oral contraceptive use and uterine leiomyoma risk: a meta-analysis based on cohort and case–control studies." Arch Gynecol Obstet288(1): 139–148.
4991	Qu, H., G. R. Sun, et al. (2013). "Clinical risk factors of delayed gastric emptying in patients after pancreaticoduodenectomy: a systematic review and meta-analysis." Eur J Surg Oncol39(3): 213–223.
5015	Rabenda, V., D. Nicolet, et al. (2013). "Relationship between use of antidepressants and risk of fractures: a meta-analysis." Osteoporos Int24(1): 121–137.
5311	Salgado, E., J. R. Maneiro, et al. (2013). "Rheumatoid factor and response to TNF antagonists in rheumatoid arthritis: Systematic review and meta-analysis of observational studies." Joint Bone Spine.
5468	Scosyrev, E., S. Tobis, et al. (2013). "Statin use and the risk of biochemical recurrence of prostate cancer after definitive local therapy: a meta-analysis of eight cohort studies." BJU Int111(3 Pt B): E71-77.
5472	Scott, I. C., R. Tan, et al. (2013). "The protective effect of alcohol on developing rheumatoid arthritis: a systematic review and meta-analysis." Rheumatology (Oxford)52(5): 856–867.
5644	Singh, I., S. Rajagopalan, et al. (2013). "Preoperative statin therapy is associated with lower requirement of renal replacement therapy in patients undergoing cardiac surgery: a meta-analysis of observational studies." Interact Cardiovasc Thorac Surg17(2): 345–352.
5646	Singh, P. P. and S. Singh (2013). "Statins are associated with reduced risk of gastric cancer: a systematic review and meta-analysis." Ann Oncol24(7): 1721–1730.
5652	Singh, S., S. K. Garg, et al. (2013). "Acid-suppressive medications and risk of oesophageal adenocarcinoma in patients with Barrett's oesophagus: a systematic review and meta-analysis." Gut.
5662	Singh, S., A. G. Singh, et al. (2013). "Statins are associated with reduced risk of esophageal cancer, particularly in patients with Barrett's esophagus: a systematic review and meta-analysis." Clin Gastroenterol Hepatol11(6): 620–629.
5674	Singh, S., P. P. Singh, et al. (2013). "Statins are associated with a reduced risk of hepatocellular cancer: a systematic review and meta-analysis." Gastroenterology144(2): 323–332.
5768	Song, Y., H. Nie, et al. (2013). "Association of statin use with risk of dementia: a meta-analysis of prospective cohort studies." Geriatr Gerontol Int13(4): 817–824.
5902	Sun, A., R. Liu, et al. (2013). "Insulin therapy and risk of colorectal cancer: an updated meta-analysis of epidemiological studies." Curr Med Res Opin.
5912	Sun, K., J. M. Liu, et al. (2013). "Bisphosphonate treatment and risk of esophageal cancer: a meta-analysis of observational studies." Osteoporos Int24(1): 279–286.
6126	Tleyjeh, I. M., A. B. Abdulhak, et al. (2013). "The association between histamine 2 receptor antagonist use and Clostridium difficile infection: a systematic review and meta-analysis." PLoS One8(3): e56498.
6148	Toulis, K. A., K. Hemming, et al. (2014). "beta-adrenergic receptor antagonists and fracture risk: a meta-analysis of selectivity, gender, and site-specific effects." Osteoporos Int25(1): 121–129.
6194	Tsakok, T., T. M. McKeever, et al. (2013). "Does early life exposure to antibiotics increase the risk of eczema? A systematic review." Br J Dermatol169(5): 983–991.
6196	Tsang, S. T. and P. Gaston (2013). "Adverse peri-operative outcomes following elective total hip replacement in diabetes mellitus: a systematic review and meta-analysis of cohort studies." Bone Joint J95-B(11): 1474–1479.
6245	Undela, K., K. Gudala, et al. (2013). "Statin use and risk of Parkinson's disease: a meta-analysis of observational studies." J Neurol260(1): 158–165.
6363	Varas-Lorenzo, C., N. Riera-Guardia, et al. (2013). "Myocardial infarction and individual nonsteroidal anti-inflammatory drugs meta-analysis of observational studies." Pharmacoepidemiol Drug Saf22(6): 559–570.
6427	Vis, M. M., M. A. Beijk, et al. (2013). "A systematic review and meta-analysis on primary percutaneous coronary intervention of an unprotected left main coronary artery culprit lesion in the setting of acute myocardial infarction." JACC Cardiovasc Interv6(4): 317–324.
6524	Wang, L., S. Cai, et al. (2013). "Insulin therapy contributes to the increased risk of colorectal cancer in diabetes patients: a meta-analysis." Diagn Pathol8(1): 180.
6587	Wang, Z. M., D. Zhao, et al. (2013). "Flavonol intake and the risk for stroke: A meta-analysis of cohort studies." Nutrition.
6761	Wu, Q., W. Qu, et al. (2013). "Tricyclic antidepressant use and risk of fractures: a meta-analysis of cohort and case–control studies." J Bone Miner Res28(4): 753–763.
6777	Wu, X. D., K. Zeng, et al. (2013). "Statins are associated with reduced risk of gastric cancer: a meta-analysis." Eur J Clin Pharmacol69(10): 1855–1860.
6801	Xie, Q., M. L. Chen, et al. (2013). "Isoflavone consumption and risk of breast cancer: a dose–response meta-analysis of observational studies." Asia Pac J Clin Nutr22(1): 118–127.
6817	Xing, D., X. L. Ma, et al. (2014). "Association between use of benzodiazepines and risk of fractures: a meta-analysis." Osteoporos Int25(1): 105–120.
99	Ahn, J. S., C. S. Eom, et al. (2013). "Acid suppressive drugs and gastric cancer: a meta-analysis of observational studies." World J Gastroenterol19(16): 2560–2568.
1479	Deshpande, A., V. Pasupuleti, et al. (2013). "Acid-suppressive therapy is associated with spontaneous bacterial peritonitis in cirrhotic patients: a meta-analysis." J Gastroenterol Hepatol28(2): 235–242.
1567	Dong, J. Y., W. G. Zhang, et al. (2013). "Vitamin D intake and risk of type 1 diabetes: a meta-analysis of observational studies." Nutrients5(9): 3551–3562.
2881	Jin, G., Z. Lanlan, et al. (2014). "Pregnancy outcome following loop electrosurgical excision procedure (LEEP) a systematic review and meta-analysis." Arch Gynecol Obstet289(1): 85–99.
6972	Yuhara, H., D. A. Corley, et al. (2013). "Aspirin and non-aspirin NSAIDs increase risk of colonic diverticular bleeding: a systematic review and meta-analysis." J Gastroenterol.
3105	Khan, M. F., C. S. Wendel, et al. (2013). "Effects of percutaneous revascularization of chronic total occlusions on clinical outcomes: a meta-analysis comparing successful versus failed percutaneous intervention for chronic total occlusion." Catheter Cardiovasc Interv82(1): 95–107.
2357	Haider, B. A., I. Olofin, et al. (2013). "Anaemia, prenatal iron use, and risk of adverse pregnancy outcomes: systematic review and meta-analysis." BMJ346: f3443.
1865	Filion, K. B., D. Chateau, et al. (2013). "Proton pump inhibitors and the risk of hospitalisation for community-acquired pneumonia: replicated cohort studies with meta-analysis." Gut.
Reason for exclusion:	
There is no comparison group (*n* = 22)	
Study ID	Reference of included citation
239	Andrews, P. L., J. R. Shiber, et al. (2013). "Early application of airway pressure release ventilation may reduce mortality in high-risk trauma patients: a systematic review of observational trauma ARDS literature." J Trauma Acute Care Surg75(4): 635–641.
2186	Gomberawalla, M. M., B. S. Miller, et al. (2013). "Meta-analysis of joint preservation versus arthroplasty for the treatment of displaced 3- and 4-part fractures of the proximal humerus." Injury44(11): 1532–1539.
2214	Gopal, M., N. Padayatchi, et al. (2013). "Systematic review of clofazimine for the treatment of drug-resistant tuberculosis." Int J Tuberc Lung Dis17(8): 1001–1007.
2749	Iftikhar, I. H., E. R. Hays, et al. (2013). "Effect of oral appliances on blood pressure in obstructive sleep apnea: a systematic review and meta-analysis." J Clin Sleep Med9(2): 165–174.
3242	Koy, A., M. Hellmich, et al. (2013). "Effects of deep brain stimulation in dyskinetic cerebral palsy: a meta-analysis." Mov Disord28(5): 647–654.
3748	Lopez-Olivo, M. A., G. Pratt, et al. (2013). "Rasburicase in tumor lysis syndrome of the adult: a systematic review and meta-analysis." Am J Kidney Dis62(3): 481–492.
4255	Moulakakis, K. G., S. N. Mylonas, et al. (2013). "A systematic review and meta-analysis of hybrid aortic arch replacement." Ann Cardiothorac Surg2(3): 247–260.
4869	Pirie, D. A., B. H. Wattar, et al. (2014). "Effects of monitoring strategies on seizures in pregnant women on lamotrigine: a meta-analysis." Eur J Obstet Gynecol Reprod Biol172: 26–31.
5866	Stone, J. C., J. Clark, et al. (2013). "Estrogen and selective estrogen receptor modulators (SERMs) for the treatment of acromegaly: a meta-analysis of published observational studies." Pituitary.
998	Chang, S. J., M. Hodeib, et al. (2013). "Survival impact of complete cytoreduction to no gross residual disease for advanced-stage ovarian cancer: a meta-analysis." Gynecol Oncol130(3): 493–498.
3796	Luksic, I., S. Clay, et al. (2013). "Effectiveness of seasonal influenza vaccines in children -- a systematic review and meta-analysis." Croat Med J54(2): 135–145.
5836	Stegeman, B. H., M. de Bastos, et al. (2013). "Different combined oral contraceptives and the risk of venous thrombosis: systematic review and network meta-analysis." BMJ347: f5298.
6128	Toczek, A., H. Cox, et al. (2013). "Strategies for reducing treatment default in drug-resistant tuberculosis: systematic review and meta-analysis." Int J Tuberc Lung Dis17(3): 299–307.
1681	El-Jurdi, N., T. Reljic, et al. (2013). "Efficacy of adoptive immunotherapy with donor lymphocyte infusion in relapsed lymphoid malignancies." Immunotherapy5(5): 457–466.
4169	Moawad, F. J., J. G. Cheatham, et al. (2013). "Meta-analysis: the safety and efficacy of dilation in eosinophilic oesophagitis." Aliment Pharmacol Ther38(7): 713–720.
5546	Sharma, S., C. Wu, et al. (2013). "Prevalence of complications in neuromuscular scoliosis surgery: a literature meta-analysis from the past 15 years." Eur Spine J22(6): 1230–1249.
6104	Tian, D. H., B. Wan, et al. (2013). "A systematic review and meta-analysis on the safety and efficacy of the frozen elephant trunk technique in aortic arch surgery." Ann Cardiothorac Surg2(5): 581–591.
449	Barrachina-Diez, J. M., E. Tashkandi, et al. (2013). "Long-term Outcome of One-Piece Implants. Part II: Prosthetic Outcomes. A Systematic Literature Review with Meta-Analysis." Int J Oral Maxillofac Implants28(6): 1470–1482.
2587	Hollingsworth, J. M., M. A. Rogers, et al. (2013). "Determining the noninfectious complications of indwelling urethral catheters: a systematic review and meta-analysis." Ann Intern Med159(6): 401–410.
2787	Iudici, M., S. Fasano, et al. (2013). "Prevalence and factors associated with glucocorticoids (GC) use in systemic sclerosis (SSc): a systematic review and meta-analysis of cohort studies and registries." Clin Rheumatol.i
4562	Orman, E. S., N. Li, et al. (2013). "Efficacy and durability of radiofrequency ablation for Barrett's Esophagus: systematic review and meta-analysis." Clin Gastroenterol Hepatol11(10): 1245–1255.
5702	Slater, N. J., M. van der Kolk, et al. (2013). "Biologic grafts for ventral hernia repair: a systematic review." Am J Surg205(2): 220–230.
Reason for exclusion:	
Not a meta-analysis/No statistical pooling of the results (*n* = 16)	
Study ID	Reference of included citation
175	Almekhlafi, M. A., B. K. Menon, et al. (2013). "A meta-analysis of observational intra-arterial stroke therapy studies using the Merci device, Penumbra system, and retrievable stents." AJNR Am J Neuroradiol34(1): 140–145.
1103	Chiappini, E., L. Galli, et al. (2013). "Use of combination neonatal prophylaxis for the prevention of mother-to-child transmission of HIV infection in European high-risk infants." AIDS27(6): 991–1000.
1505	Dienstknecht, T., K. Horst, et al. (2013). "A meta-analysis of operative versus nonoperative treatment in 463 scapular neck fractures." Scand J Surg102(2): 69–76.
2431	Hasan, S., L. Liu, et al. (2013). "The role of postoperative radiation and chemoradiation in merkel cell carcinoma: a systematic review of the literature." Front Oncol3: 276.
1641	Eckert, S., S. B. Freytag, et al. (2013). "Meta-analysis of three observational studies of amlodipine/valsartan in hypertensive patients with additional risk factors." Blood Press22 Suppl 1: 11–21.
960	Chacko, J., L. Harling, et al. (2013). "Can statins improve outcomes after isolated cardiac valve surgery? A systematic literature review." Clin Cardiol36(8): 448–455.
3376	Lane, D. A., S. Raichand, et al. (2013). "Combined anticoagulation and antiplatelet therapy for high-risk patients with atrial fibrillation: a systematic review." Health Technol Assess17(30): 1–188.
1926	Forman-Hoffman, V. L., A. J. Zolotor, et al. (2013). "Comparative effectiveness of interventions for children exposed to nonrelational traumatic events." Pediatrics131(3): 526–539.
141	Alcoba, G., M. Kerac, et al. (2013). "Do children with uncomplicated severe acute malnutrition need antibiotics? A systematic review and meta-analysis." PLoS One8(1): e53184.
2763	Ingargiola, M. J., L. N. Daniali, et al. (2013). "Does the application of incisional negative pressure therapy to high-risk wounds prevent surgical site complications? A systematic review." Eplasty13: e49.
3250	Krag, M., A. Perner, et al. (2013). "Stress ulcer prophylaxis in the intensive care unit: is it indicated? A topical systematic review." Acta Anaesthesiol Scand57(7): 835–847.
4889	Polk, A., M. Vaage-Nilsen, et al. (2013). "Cardiotoxicity in cancer patients treated with 5-fluorouracil or capecitabine: a systematic review of incidence, manifestations and predisposing factors." Cancer Treat Rev39(8): 974–984.
4941	Proietti, R., G. M. Manzoni, et al. (2013). "Can Cardiac Resynchronization Therapy Improve Cognitive Function? A Systematic Review." Pacing Clin Electrophysiol.
1013	Chapelle, C., S. Quenet, et al. (2013). "Antipsychotics: a real or confounding risk factor for venous thromboembolism?" Pharmacopsychiatry46(1): 36–37.
1395	de Tayrac, R. and L. Sentilhes (2013). "Complications of pelvic organ prolapse surgery and methods of prevention." Int Urogynecol J24(11): 1859–1872.
1559	Domecq, J. P., G. Prutsky, et al. (2013). "Adverse effects of the common treatments for polycystic ovary syndrome: a systematic review and meta-analysis." J Clin Endocrinol Metab98(12): 4646–4654.
Reason for exclusion:	
Meta-Analysis does not include results from observational studies (*n* = 12)	
Study ID	Reference of included citation
87	Ahmed, F., M. C. Lindley, et al. (2014). "Effect of influenza vaccination of healthcare personnel on morbidity and mortality among patients: systematic review and grading of evidence." Clin Infect Dis58(1): 50–57.
125	Albanna, A. S., B. M. Smith, et al. (2013). "Fixed-dose combination antituberculosis therapy: a systematic review and meta-analysis." Eur Respir J42(3): 721–732.
1982	Fu, R., S. Selph, et al. (2013). "Effectiveness and harms of recombinant human bone morphogenetic protein-2 in spine fusion: a systematic review and meta-analysis." Ann Intern Med158(12): 890–902.
3004	Kansagara, D., E. Dyer, et al. (2013). "Treatment of anemia in patients with heart disease: a systematic review." Ann Intern Med159(11): 746–757.
2525	Henson, C. C., S. Burden, et al. (2013). "Nutritional interventions for reducing gastrointestinal toxicity in adults undergoing radical pelvic radiotherapy." Cochrane Database Syst Rev11: CD009896.
6034	Taylor, P. N., O. E. Okosieme, et al. (2014). "Therapy of Endocrine Disease: Impact of iodine supplementation in mild-to-moderate iodine deficiency: systematic review and meta-analysis." Eur J Endocrinol170(1): R1-R15.
753	Bredemeier, M., F. K. de Oliveira, et al. (2013). "Low- versus high-dose rituximab for rheumatoid arthritis: A systematic review and meta-analysis." Arthritis Care Res (Hoboken).
1791	Fandino, M., K. I. Macdonald, et al. (2013). "The use of postoperative topical corticosteroids in chronic rhinosinusitis with nasal polyps: a systematic review and meta-analysis." Am J Rhinol Allergy27(5): e146-157.
3316	Kwok, C. S., V. Jeevanantham, et al. (2013). "No consistent evidence of differential cardiovascular risk amongst proton-pump inhibitors when used with clopidogrel: meta-analysis." Int J Cardiol167(3): 965–974.
3012	Kapadia, S., S. Hapani, et al. (2013). "Risk of liver toxicity with the angiogenesis inhibitor pazopanib in cancer patients." Acta Oncol52(6): 1202–1212.
5089	Reichenpfader, U., G. Gartlehner, et al. (2013). "Sexual Dysfunction associated with Second-Generation Antidepressants in Patients with Major Depressive Disorder: Results from a Systematic Review with Network Meta-Analysis." Drug Saf.
1936	Fortmann, S. P., B. U. Burda, et al. (2013). "Vitamin and Mineral Supplements in the Primary Prevention of Cardiovascular Disease and Cancer: An Updated Systematic Evidence Review for the U.S. Preventive Services Task Force." Ann Intern Med.
Reason for exclusion:	
Full text not available (*n* = 10)	
Study ID	Reference of included citation
3702	Liu, Y., P. Su, et al. (2013). "Endoscopic sphincterotomy plus balloon dilation versus endoscopic sphincterotomy for choledocholithiasis: A meta-analysis." J Gastroenterol Hepatol28(6): 937–945.
3704	Liu, Y., W. Tang, et al. (2013). "Association between statin use and colorectal cancer risk: a meta-analysis of 42 studies." Cancer Causes Control.
4701	Pasipanodya, J. G. and T. Gumbo (2013). "A meta-analysis of self-administered vs directly observed therapy effect on microbiologic failure, relapse, and acquired drug resistance in tuberculosis patients." Clin Infect Dis57(1): 21–31.
5285	Saheb, K. J., B. Q. Deng, et al. (2013). "Triple antithrombotic therapy versus double antiplatelet therapy after percutaneous coronary intervention with stent implantation in patients requiring chronic oral anticoagulation: a meta-analysis." Chin Med J (Engl)126(13): 2536–2542.
5922	Sun, Z. J., W. J. Li, et al. (2013). "Comparing minimally invasive and open transforaminal lumbar interbody fusion for treatment of degenerative lumbar disease: a meta-analysis." Chin Med J (Engl)126(20): 3962–3971.
6783	Wu, Y. C., J. F. Zhang, et al. (2013). "Transcatheter aortic valve implantation versus surgical aortic valve replacement for severe aortic stenosis: a meta analysis." Chin Med J (Engl)126(6): 1171–1177.
7106	Zheng, Z., L. Sheng, et al. (2013). "Statins and amyotrophic lateral sclerosis: a systematic review and meta-analysis." Amyotroph Lateral Scler Frontotemporal Degener14(4): 241–245.
3598	Lin, G. H., H. L. Chan, et al. (2013). "The Effect of Flapless Surgery on Implant Survival and Marginal Bone Level: A Systematic Review and Meta-analysis." J Periodontol.
4225	More, K., G. Athalye-Jape, et al. (2013). "Association of inhibitors of gastric acid secretion and higher incidence of necrotizing enterocolitis in preterm very low-birth-weight infants." Am J Perinatol30(10): 849–856.
4175	Moen, C. A., A. Burrell, et al. (2013). "Does tranexamic acid stop haemoptysis?" Interact Cardiovasc Thorac Surg17(6): 991–994.
Reason for exclusion:	
Meta-analysis not main objective (*n* = 6)	
Study ID	Reference of included citation
1469	D'Errigo, P., F. Biancari, et al. (2013). "Thirty-day mortality after coronary artery bypass surgery in patients aged <50 years: results of a multicenter study and meta-analysis of the literature." J Card Surg28(3): 207–211.
1735	Etminan, M., S. T. Bird, et al. (2013). "Isotretinoin and risk for inflammatory bowel disease: a nested case–control study and meta-analysis of published and unpublished data." JAMA Dermatol149(2): 216–220.
1753	Faggioli, G., R. Pini, et al. (2013). "Contralateral carotid occlusion in endovascular and surgical carotid revascularization: a single centre experience with literature review and meta-analysis." Eur J Vasc Endovasc Surg46(1): 10–20.
3067	Kennedy, N. A., E. Rhatigan, et al. (2013). "A trial of mercaptopurine is a safe strategy in patients with inflammatory bowel disease intolerant to azathioprine: an observational study, systematic review and meta-analysis." Aliment Pharmacol Ther38(10): 1255–1266.
3624	Linehan, M. F., U. Nurmatov, et al. (2013). "Does BCG vaccination protect against childhood asthma? Final results from the Manchester Community Asthma Study retrospective cohort study and updated systematic review and meta-analysis." J Allergy Clin Immunol.
4455	Nouri, K., M. Demmel, et al. (2013). "Prospective cohort study and meta-analysis of cyclic bleeding after laparoscopic supracervical hysterectomy." Int J Gynaecol Obstet122(2): 124–127.
Reason for exclusion:	
No observational studies (*n* = 4)	
Study ID	Reference of included citation
946	Cates, C. J. and B. H. Rowe (2013). "Vaccines for preventing influenza in people with asthma." Cochrane Database Syst Rev2: CD000364.
2240	Gouya, G., J. Arrich, et al. (2013). "Antiplatelet Treatment for Prevention of Cerebrovascular Events in Patients With Vascular Diseases: A Systematic Review and Meta-Analysis." Stroke.
6484	Wang, C. H., C. H. Huang, et al. (2013). "Biphasic versus monophasic defibrillation in out-of-hospital cardiac arrest: a systematic review and meta-analysis." Am J Emerg Med31(10): 1472–1478.
4859	Pineda, A. M., F. O. Nascimento, et al. (2013). "A meta-analysis of transcatheter closure of patent foramen ovale versus medical therapy for prevention of recurrent thromboembolic events in patients with cryptogenic cerebrovascular events." Catheter Cardiovasc Interv.
Reason for exclusion:	
Not published in 2013 (*n* = 4)	
Study ID	Reference of included citation
179	Almenawer, S. A., F. Farrokhyar, et al. (2013). "Chronic Subdural Hematoma Management: A Systematic Review and Meta-analysis of 34829 Patients." Ann Surg.
3159	Klatte, T., S. F. Shariat, et al. (2013). "Systematic review and meta-analysis of perioperative and oncological outcomes of laparoscopic cryoablation versus laparoscopic partial nephrectomy for the treatment of small renal tumors." J Urol.
5708	Sligl, W. I., L. Asadi, et al. (2013). "Macrolides and Mortality in Critically Ill Patients With Community-Acquired Pneumonia: A Systematic Review and Meta-Analysis." Crit Care Med.
6482	Wang, C. H., W. H. Hsieh, et al. (2013). "Liberal Versus Restricted Fluid Resuscitation Strategies in Trauma Patients: A Systematic Review and Meta-Analysis of Randomized Controlled Trials and Observational Studies." Crit Care Med.
Reason for exclusion:	
Individual Patient Data meta-analysis (*n* = 2)	
Study ID	Reference of included citation
5616	Simmonds, M. C., J. V. Brown, et al. (2013). "Safety and effectiveness of recombinant human bone morphogenetic protein-2 for spinal fusion: a meta-analysis of individual-participant data." Ann Intern Med158(12): 877–889.
485	Bazelier, M. T., F. de Vries, et al. (2013). "Risk of fracture with thiazolidinediones: an individual patient data meta-analysis." Front Endocrinol (Lausanne)4: 11.

## Abbreviations

CER: comparative effectiveness research
NRSI: non-randomized studies of interventions
RCT: randomized controlled trial
RoB Tool: Risk of Bias tool

## References

[1] Castillo RC, Scharfstein DO, MacKenzie EJ (2012). Observational studies in the era of randomized trials: finding the balance. J Bone Joint Surg Am.

[2] Chou R, Helfand M (2005). Challenges in systematic reviews that assess treatment harms. Ann Intern Med.

[3] Grootendorst DC, Jager KJ, Zoccali C, Dekker FW (2010). Observational studies are complementary to randomized controlled trials. Nephron.

[4] Yang W, Zilov A, Soewondo P, Bech OM, Sekkal F, Home PD (2010). Observational studies: going beyond the boundaries of randomized controlled trials. Diabetes Res Clin Pract.

[5] Black N (1996). Why we need observational studies to evaluate the effectiveness of health care. Bmj.

[6] The Cochrane Collaboration Glossary [http://www.cochrane.org/glossary/5#letters]

[7] Benson K, Hartz AJ (2000). A comparison of observational studies and randomized, controlled trials. N Engl J Med.

[8] Goulart BH, Ramsey SD, Parvathaneni U (2014). Observational study designs for comparative effectiveness research: an alternative approach to close evidence gaps in head-and-neck cancer. Int J Radiat Oncol Biol Phys.

[9] Silverman SL (2009). From randomized controlled trials to observational studies. Am J Med.

[10] Hannan EL (2008). Randomized clinical trials and observational studies: guidelines for assessing respective strengths and limitations. JACC Cardiovasc Interv.

[11] Alemayehu D, Cappelleri JC (2013). Revisiting issues, drawbacks and opportunities with observational studies in comparative effectiveness research. J Eval Clin Pract.

[12] Garabedian LF, Chu P, Toh S, Zaslavsky AM, Soumerai SB (2014). Potential bias of instrumental variable analyses for observational comparative effectiveness research. Ann Intern Med.

[13] Marko NF, Weil RJ (2010). The role of observational investigations in comparative effectiveness research. Value Health.

[14] Roche N, Reddel H, Martin R, Brusselle G, Papi A, Thomas M (2014). Quality standards for real-world research. Focus on observational database studies of comparative effectiveness. Ann Am Thorac Soc.

[15] Shrier I, Boivin JF, Steele RJ, Platt RW, Furlan A, Kakuma R (2007). Should meta-analyses of interventions include observational studies in addition to randomized controlled trials? A critical examination of underlying principles. Am J Epidemiol.

[16] Norris SL, Atkins D, Bruening W, Fox S, Johnson E, Kane R (2011). Observational studies in systematic [corrected] reviews of comparative effectiveness: AHRQ and the Effective Health Care Program. J Clin Epidemiol.

[17] Holve E, Pittman P (2009). A first look at the volume and cost of comparative effectiveness research in the United States [monograph].

[18] Abraham NS, Byrne CJ, Young JM, Solomon MJ (2010). Meta-analysis of well-designed nonrandomized comparative studies of surgical procedures is as good as randomized controlled trials. Journal of Clinical Epidemiology.

[19] Concato J, Shah N, Horwitz RI (2000). Randomized, controlled trials, observational studies, and the hierarchy of research designs. N Engl J Med.

[20] Golder S, Loke YK, Bland M (2011). Meta-analyses of adverse effects data derived from randomised controlled trials as compared to observational studies: methodological overview. PLoS Med.

[21] Ioannidis JP, Haidich AB, Pappa M, Pantazis N, Kokori SI, Tektonidou MG (2001). Comparison of evidence of treatment effects in randomized and nonrandomized studies. Jama.

[22] Vandenbroucke JP (2004). When are observational studies as credible as randomised trials?. Lancet.

[23] Zhang Z, Ni H, Xu X (2014). Observational studies using propensity score analysis underestimated the effect sizes in critical care medicine. J Clin Epidemiol.

[24] Higgins JPT, Green S, editors: *Cochrane Handbook for Systematic Reviews of Interventions* Version 5.1.0 [updated March 2011]. *The Cochrane Collaboration* 2011, Available from www.cochrane-handbook.org

[25] Brugha TS, Matthews R, Morgan Z, Hill T, Alonso J, Jones DR (2012). Methodology and reporting of systematic reviews and meta-analyses of observational studies in psychiatric epidemiology: systematic review. Br J Psychiatry.

[26] Golder S, Loke Y, McIntosh HM (2006). Room for improvement?. A survey of the methods used in systematic reviews of adverse effects. BMC Med Res Methodol.

[27] Moher D, Tetzlaff J, Tricco AC, Sampson M, Altman DG (2007). Epidemiology and reporting characteristics of systematic reviews. PLoS Med.

[28] Zorzela L, Golder S, Liu Y, Pilkington K, Hartling L, Joffe A (2014). Quality of reporting in systematic reviews of adverse events: systematic review. Bmj.

[29] Stroup DF, Berlin JA, Morton SC, Olkin I, Williamson GD, Rennie D (2000). Meta-analysis of observational studies in epidemiology: a proposal for reporting. Meta-analysis Of Observational Studies in Epidemiology (MOOSE) group. Jama.

[30] Liberati A, Altman DG, Tetzlaff J, Mulrow C, Gotzsche PC, Ioannidis JPA (2009). The PRISMA statement for reporting systematic reviews and meta-analyses of studies that evaluate health care interventions: explanation and elaboration. Journal of Clinical Epidemiology.

[31] Moher D, Liberati A, Tetzlaff J, Altman DG, Group P (2009). Preferred reporting items for systematic reviews and meta-analyses: the PRISMA Statement. Open Med.

[32] Shea BJ, Grimshaw JM, Wells GA, Boers M, Andersson N, Hamel C (2007). Development of AMSTAR: a measurement tool to assess the methodological quality of systematic reviews. BMC Med Res Methodol.

[33] Delgado-Rodriguez M, Ruiz-Canela M, De Irala-Estevez J, Llorca J, Martinez-Gonzalez A (2001). Participation of epidemiologists and/or biostatisticians and methodological quality of published controlled clinical trials. J Epidemiol Community Health.

[34] Vale CL, Tierney JF, Burdett S (2013). Can trial quality be reliably assessed from published reports of cancer trials: evaluation of risk of bias assessments in systematic reviews. Bmj.

[35] Murad MH, Montori VM, Ioannidis JP, Jaeschke R, Devereaux PJ, Prasad K (2014). How to read a systematic review and meta-analysis and apply the results to patient care: users' guides to the medical literature. Jama.

[36] Dal-Re R, Ioannidis JP, Bracken MB, Buffler PA, Chan AW, Franco EL (2014). Making prospective registration of observational research a reality. Sci Transl Med.

[37] Baudart M, Ravaud P, Baron G, Dechartres A, Haneef R, Boutron I (2016). Public availability of results of observational studies evaluating an intervention registered at ClinicalTrials.gov. BMC Med.

[38] Ioannidis JP, Trikalinos TA (2007). The appropriateness of asymmetry tests for publication bias in meta-analyses: a large survey. Cmaj.

[39] Terrin N, Schmid CH, Lau J (2005). In an empirical evaluation of the funnel plot, researchers could not visually identify publication bias. J Clin Epidemiol.

[40] Hopewell S, Boutron I, Altman DG, Ravaud P (2013). Incorporation of assessments of risk of bias of primary studies in systematic reviews of randomised trials: a cross-sectional study. BMJ Open.

[41] Higgins JP, Altman DG, Gotzsche PC, Juni P, Moher D, Oxman AD (2011). The Cochrane Collaboration’s tool for assessing risk of bias in randomised trials. Bmj.

[42] Jadad AR, Moore RA, Carroll D, Jenkinson C, Reynolds DJ, Gavaghan DJ (1996). Assessing the quality of reports of randomized clinical trials: is blinding necessary?. Control Clin Trials.

[43] Atkins D, Best D, Briss PA, Eccles M, Falck-Ytter Y, Flottorp S (2004). Grading quality of evidence and strength of recommendations. Bmj.

[44] Wells GA, Shea BJ, O'Connell D, Peterson J, Welch V, Losos M et al*.* The Newcastle-Ottawa Scale (NOS) for assessing the quality of nonrandomised studies in meta-analyses. http://www.ohri.ca/programs/clinical_epidemiology/oxford.asp. 2013.

